# Oxali(IV)Fluors: Fluorescence Responsive Oxaliplatin(IV) Complexes Identify a Hypoxia-Dependent Reduction in Cancer Cells

**DOI:** 10.1021/jacs.3c03320

**Published:** 2023-06-07

**Authors:** Marie H. C. Boulet, Hannah R. Bolland, Ester M. Hammond, Adam C. Sedgwick

**Affiliations:** † Chemistry Research Laboratory, Department of Chemistry, University of Oxford, Mansfield Road, Oxford, OX1 3TA, United Kingdom; ‡ Department of Oncology, University of Oxford, Old Road Campus Research Building, Oxford, OX3 7DQ, United Kingdom

## Abstract

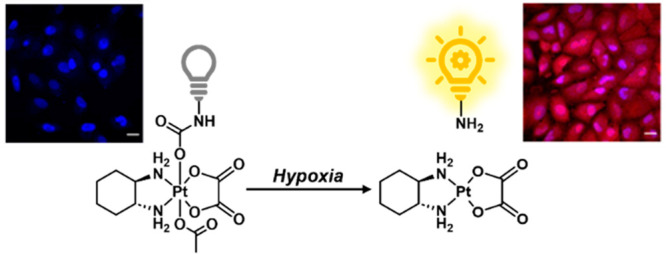

Platinum(IV) anticancer agents have demonstrated the potential to overcome the limitations associated with the widely used Pt(II) chemotherapeutics, cisplatin, carboplatin, and oxaliplatin. In order to identify therapeutic scenarios where this type of chemotherapy can be applied, an improved understanding on the intracellular reduction of Pt(IV) complexes is needed. Here, we report the synthesis of two fluorescence responsive oxaliplatin(IV)(OxPt) complexes, OxaliRes and OxaliNap. Sodium ascorbate (NaAsc) was shown to reduce each OxPt(IV) complex resulting in increases in their respective fluorescence emission intensities at 585 and 545 nm. The incubation of each OxPt(IV) complex with a colorectal cancer cell line resulted in minimal changes to the respective fluorescence emission intensities. In contrast, the treatment of these cells with NaAsc showed a dose-dependent increase in fluorescence emission intensity. With this knowledge in hand, we tested the reducing potential of tumor hypoxia, where an oxygen-dependent bioreduction was observed for each OxPt(IV) complex with <0.1% O_2_ providing the greatest fluorescence signal. Clonogenic cell survival assays correlated with these observations demonstrating significant differences in toxicity between hypoxia (<0.1% O_2_) and normoxia (21% O_2_). To the best of our knowledge, this is the first report showing carbamate-functionalized OxPt(IV) complexes as potential hypoxia-activated prodrugs.

Pt(IV) anticancer agents have emerged as attractive alternatives to the FDA-approved Pt(II) therapeutics, cisplatin, carboplatin, and oxaliplatin.^1−4^ These six-coordinate octahedral Pt(IV) complexes, with two additional ligands in the axial positions are kinetically more inert than their Pt(II) precursors, which minimalizes off-target interactions. Thus, reducing side effects, and enhancing bioavailability.^2^ Intracellular reduction is thought to afford the cytotoxic Pt(II) complex and release the two axial ligands.^1,5−7^ Despite promising clinical studies, no Pt(IV) anticancer agent, has been clinically approved to date. Improving our understanding on how and when these Pt(IV) therapeutics are activated will help identify clinical scenarios for the successful implementation of this type of therapy.^8^ In an effort to elucidate the reduction mechanism, we report two fluorescence responsive oxaliplatin(IV) complexes, OxaliFluors (OxaliRes and OxaliNap), that enable the visualization of Pt(IV) reduction in cells (Scheme 1).

**Scheme 1:**
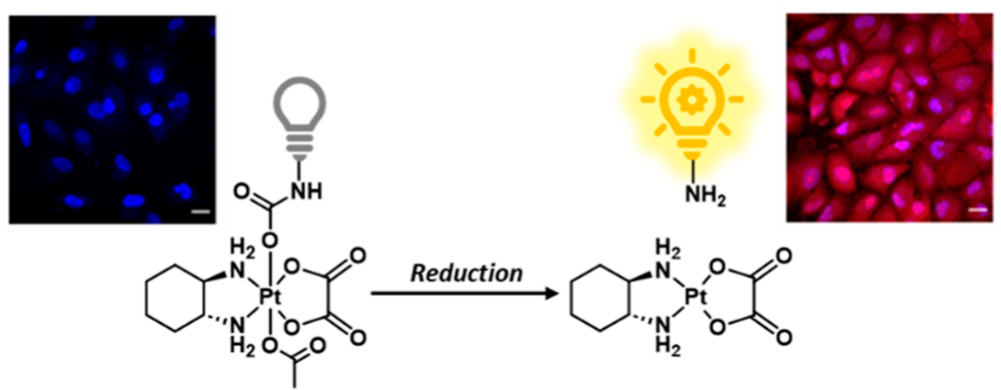
Basic Schematic of the OxaliFluor Strategy

High performance liquid chromatography (HPLC) analysis is the current gold standard for evaluating Pt(IV) reduction.^6^ Although crucial for determining the reduction profiles of Pt(IV) complexes with biological reductants (e.g., sodium ascorbate, NaAsc), HPLC is limited to solution-based studies.^6^ Fluorescence probe strategies provide a powerful alternative to allow the noninvasive study of Pt-chemistry in cells.^9,10^ Early fluorescence probe examples developed by the groups of New, Hambley, Leung, and Ang focused on chelation-based strategies for the detection of Pt(II) ions in solution and in cells.^11−13^ Such systems were shown capable of indirectly monitoring cisplatin(IV) reduction.^14^ Other reported strategies incorporated fluorophores onto the equatorial or axial positions of Pt(IV) complexes to directly measure Pt(IV) reduction.^15−19^ In a recent report, Zhu and co-workers incorporated a BODIPY fluorophore on the axial position of a carboplatin Pt(IV) analogue to study intracellular reduction. However, despite this report and others, minimal information on the factors that lead to intracellular reduction is available.^15^ Here, we rationalized by incorporating amino-based fluorophores onto the axial position of Pt(IV) complexes, we would readily achieve an “off” to “on” fluorescence response upon reduction in cancer cells. To test this hypothesis, we synthesized two fluorescent complexes, OxaliRes and OxaliNap using oxaliplatin(IV)(OxPt) as the model Pt(IV) complex and resorufin (Res) and naphthalimide (Nap) as the respective fluorophores. Each complex differed by how they were linked to the corresponding fluorophore to permit a direct head-to-head comparison between the fluorescence probe design of “direct attachment” vs “self-immolative linker”^17^ (Figure 1). The full synthetic procedures and characterization of OxaliRes and OxaliNap can be found in the Supporting Information (SI) (Schemes S1–S4). Purities of >95% were determined by LC-MS analysis (see SI, Figures S1–S2).

**Figure 1:**
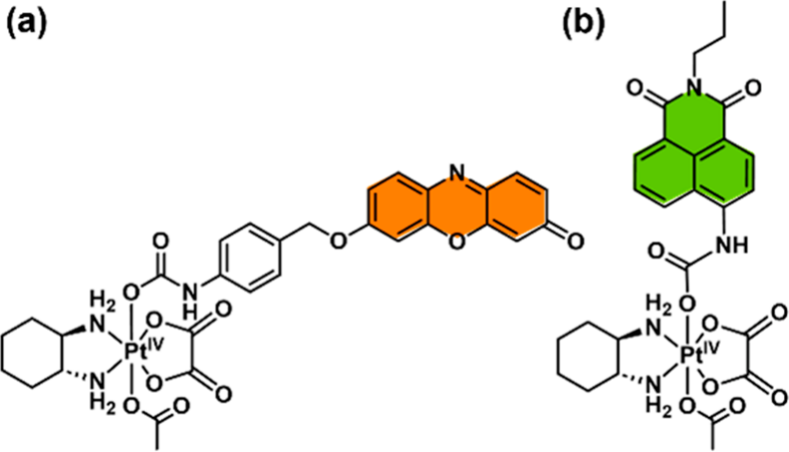
(a) Chemical structure of OxaliRes. (b) Chemical structure of OxaliNap.

With both OxaliRes and OxaliNap in hand, we turned our attention to evaluating the photophysical and chemical properties of each complex in aqueous solution (PBS, pH = 7.40). As expected, OxaliRes and OxaliNap displayed NaAsc-dependent increases in fluorescence emission intensities at 585 and 545 nm, respectively, indicative of the reduction of OxPt(IV) to OxPt(II) (Figure 2a and 2b). Noticeable changes in the absorption spectra were observed consistent with the release of the respective fluorophores, resorufin and 4-NH_2_-Nap (**5**) (see SI, Figures S3–S4). To observe changes in fluorescence intensities, incubation times (>1 h) were required (see SI, Figures S5–S6). Interestingly, differences in NaAsc sensitivity were seen between each complex. OxaliRes responded to concentration ranges between 0 and 4 mM NaAsc, whereas OxaliNap relied on >5 mM to achieve at least a 4-fold increase in fluorescence intensity (Figure 2a and 2b). To rationalize this observation, cyclic voltammetry experiments were performed with OxPt(OH)(OAc) (**1**), OxaliRes, and OxaliNap (see SI, Figures S7–S9). The more positive reduction peak (*E*_*p*_) of the resorufin ligand (*E*_*p*_ = −1.20 vs Fc^0/+^) compared to **1** (*E_p_* = −1.88 vs Fc^0/+^) and the 4-NH_2_-Nap ligand (**5**) (*E*_*p*_ = −2.03 vs Fc^0/+^) suggests the axial ligand may influence the rate of Pt(IV) chemical reduction. Fluorescence spectroscopy and HPLC showed good stability for OxaliRes and OxaliNap over 12 h (Figure 2c and 2d; see SI, Figures S10 and S11). The increases in fluorescence emissions were found to be selective to the biological reductants NaAsc and NADH. Negligible changes were seen in the presence of other biological reductants such as l-glutathione (GSH;^20^ 4 mM) (see SI, Figures S12–S14). A spectral shift in emission wavelength was observed in the presence of BSA for OxaliNap suggesting potential protein binding^21^ (see SI, Figure S15). HPLC and LC-MS analysis confirmed the proposed mechanism with the release of each fluorophore and the reduction of Pt(IV) to Pt(II) (see SI, Figures S16–19).

**Figure 2:**
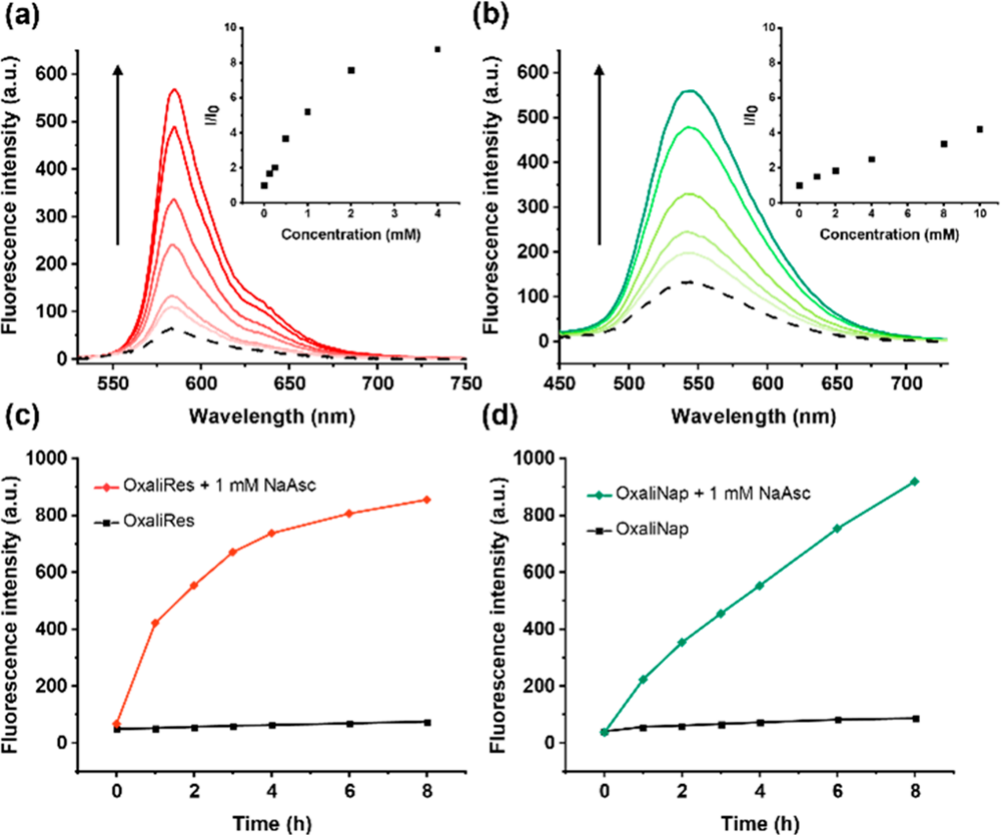
(a) Fluorescence spectra of OxaliRes (5 μM) with increasing concentrations of NaAsc (1 h incubation) λ_ex_ = 500 nm. Slit widths: ex = 10 nm, em = 2.5 nm. Inset: Relative fluorescence changes (*I*/*I*_0_) at 585 nm as a function of NaAsc concentration (0–4 mM) (b) Fluorescence spectra of OxaliNap (5 μM) with increasing concentrations of NaAsc (1 h incubation) λ_ex_ = 430 nm. Slit widths: ex = 10 nm, em = 10 nm. Inset: Relative fluorescence changes (*I*/*I*_0_) at 545 nm as a function of NaAsc concentration (0–10 mM). (c) Changes in the fluorescence intensity of OxaliRes (5 μM) at 585 nm with (red) and without (black) the addition of NaAsc (1 mM) (d) Changes in the fluorescence intensity of OxaliNap (5 μM) at 545 nm with (green) and without (black) the addition of NaAsc (1 mM). All measurement were performed in PBS (pH = 7.40).

To date, it is unclear when OxPt(IV)-based complexes are reduced in cells. For these reasons, we pursued evaluating OxaliRes and OxaliNap in cancer cells. A colorectal HCT116 cancer cell line was chosen for testing due to the routine clinical use of OxPt(II) for the treatment of colon cancer.^22^ HCT116 cells were treated with OxaliRes or OxaliNap in the presence of NaAsc and visualized by microscopy. In the absence of NaAsc minimal changes in fluorescence emission intensities were observed. However, increasing NaAsc concentration resulted in dose-dependent increases in fluorescence emission intensities in their respective red and green emission channels (Figure 3a and 3b). Flow cytometry quantified the increases in fluorescence emission (Figure 3c and 3d). Notably, differences in NaAsc sensitivity between OxaliRes and OxaliNap previously seen in solution translated to this cell imaging study. OxaliNap required longer incubation times (120 min) and greater concentrations of NaAsc. Nevertheless, the obtained data show that (1) HCT116 cells alone are not reducing enough to reduce these carbamate-functionalized OxPt(IV) complexes and (2) excess reducing agents such as NaAsc and NADH are required to reduce OxPt(IV) to OxPt(II) in HCT116 cells.

**Figure 3:**
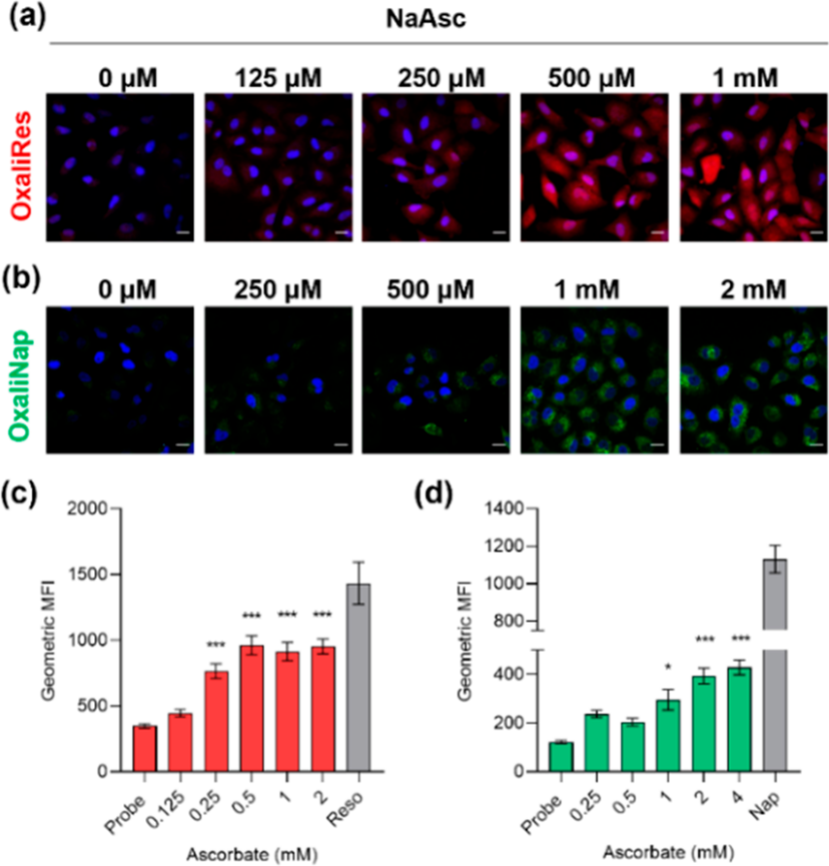
(a) Representative fluorescence images of HCT116 cells treated with OxaliRes (10 μM) with NaAsc (0–1 mM). Cells were fixed after 80 min. (b) Representative fluorescence images of HCT116 cells treated with OxaliNap (10 μM) with NaAsc (0–2 mM). Cells were fixed after 120 min. Scale bar in (a) and (b) represents 20 μM and blue color represents DAPI stain. (c, d) Flow cytometry analysis of HCT116 cells treated with OxaliRes (10 μM) or OxaliNap (10 μM) with the indicated concentrations of NaAsc. Relative fluorescence is displayed as geometric mean intensity (MFI). Error bars represent SD. Significance compared to probe alone condition. * *p* < 0.05, ** *p* < 0.01, and *** *p* < 0.001. *n* = 3.

Regions of hypoxia occur in most solid tumors as a result of inefficient vasculature and the high metabolic demand of cancer cells.^23^ Due to its correlation to chemo- and radiotherapy resistance,^24,25^ extensive efforts have been devoted to exploiting the bioreductive nature of tumor hypoxia to catalyze a wide range of biotransformations for diagnostic and therapeutic purposes.^26−28^ However, few studies have focused on the hypoxia-mediated bioreduction of Pt(IV) to Pt(II) nor its direct visualization to understand the differences between reduction and toxicity.^29−32^ We therefore set out to test OxaliRes and OxaliNap in hypoxic conditions. To start, HCT116 cells were exposed to severe levels of hypoxia (<0.1% O_2_) in the presence of OxaliRes or OxaliNap. A time-dependent increase in fluorescence emission intensities over the course of 6 h was observed suggesting that reduction of OxPt(IV) to OxPt(II) does indeed occur in hypoxia (see SI, Figure S20). However, within tumors a gradient of hypoxic conditions occurs and therefore it was important to determine the oxygen dependency of OxPt(IV) reduction in OxaliRes and OxaliNap.^25^ HCT116 cells were treated with OxaliRes and OxaliNap in a range of oxygen levels and changes in fluorescence observed by microscopy and quantified by flow cytometry. An oxygen-dependent increase in fluorescence of both OxaliRes and OxaliNap was observed in hypoxia with changes in OxaliRes significantly increasing below 4% O_2_ and OxaliNap below 1.5% O_2_ (Figure 4). Thus, highlighting a potential correlation between NaAsc sensitivity and oxygen dependency. Together, the obtained data suggest the hypoxia-mediated reduction of OxPt(IV) to OxPt(II) in HCT-116 cancer cells.

**Figure 4:**
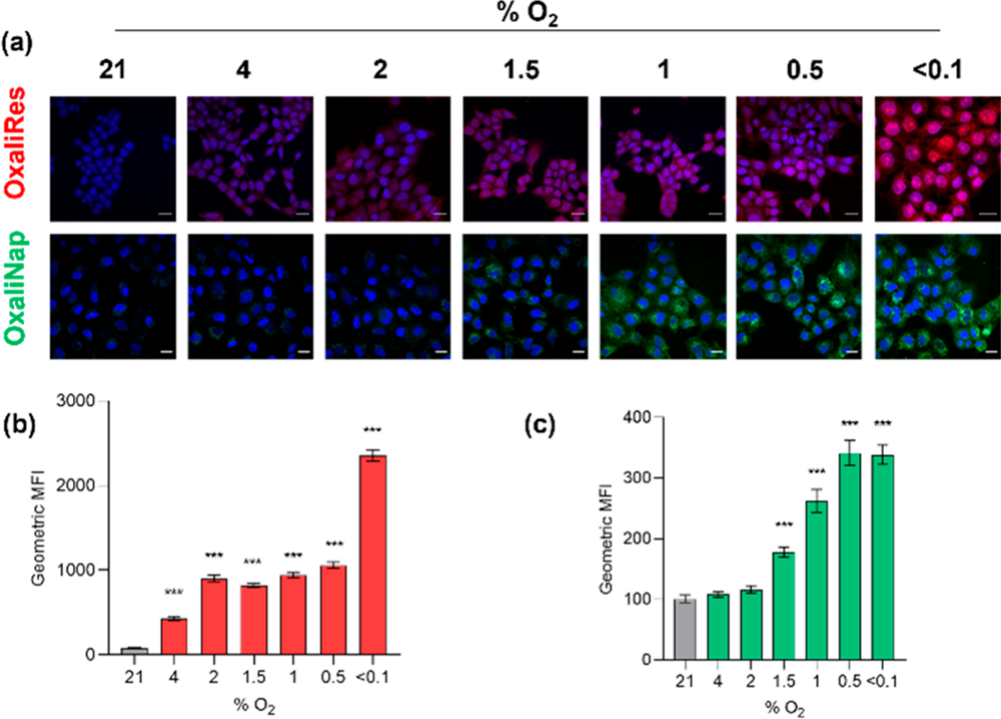
(a) Representative fluorescence images of HCT116 cells treated with OxaliNap or OxaliRes (10 μM) for 6 h in the oxygen conditions indicated. Scale bar represents 20 μM and blue color represents DAPI stain. (b, c) Flow cytometry of HCT116 cells treated with OxaliRes (10 μM) or OxaliNap (10 μM) for 6 h at the oxygen level indicated. Relative fluorescence is displayed as geometric mean intensity (MFI). Error bars represent SD. Significance compared to normoxic control. * *p* < 0.05, ** *p* < 0.01, and *** *p* < 0.001. *n* = 3.

Next, we wanted to determine if the observed hypoxic-mediated activation of OxaliRes and OxaliNap led to an increase in cytotoxicity due to the reduction of OxPt(IV) to OxPt(II). Using clonogenic cells survival assays we first confirmed that the fluorophores, resorufin, and 4-NH_2_Nap (**5**) were nontoxic to HCT116 cells (see SI, Figure S21). We then compared the cell survival of HCT116 cells treated with OxaliRes and OxaliNap in normoxic conditions (>21% O_2_) and hypoxic conditions (<0.1% O_2_). Both OxaliRes and OxaliNap were significantly more toxic in hypoxic conditions compared to normoxic conditions (Figure 5). Comparing the obtained imaging data to cytotoxicities showed that although OxaliNap proved least sensitive to reduction, a greater toxicity in normoxic conditions was observed, suggesting initial cytotoxicity from the Pt(IV) species. OxPt(IV)(OH)(OAc) (**1**) showed smaller differences in cytotoxicity between normoxic and hypoxic conditions, and as expected, OxPt(II) displayed the greatest cytotoxicity and was equally toxic in normoxia and hypoxia (see SI, Figure S22). Cytotoxicity in another colorectal cell line, RKO, showed similar results (see SI, Figures S23–S24). 4-Aminobenzyl alcohol, a side product from OxaliRes, displayed no toxicity (see SI, Figure S25). Overall, we believe these data indicate that the carbamate functionality on OxPt(IV) can dictate the initial cytotoxicity of the Pt(IV) species and the sensitivity of each Pt(IV) species to reduction is an important factor to study to enable the differentiation between toxicities in normoxic and hypoxic conditions. We believe this fluorescent strategy has provided insight into new potential design strategies for OxPt(IV)-based prodrugs, and tumor hypoxia has been identified as a therapeutic target.

**Figure 5:**
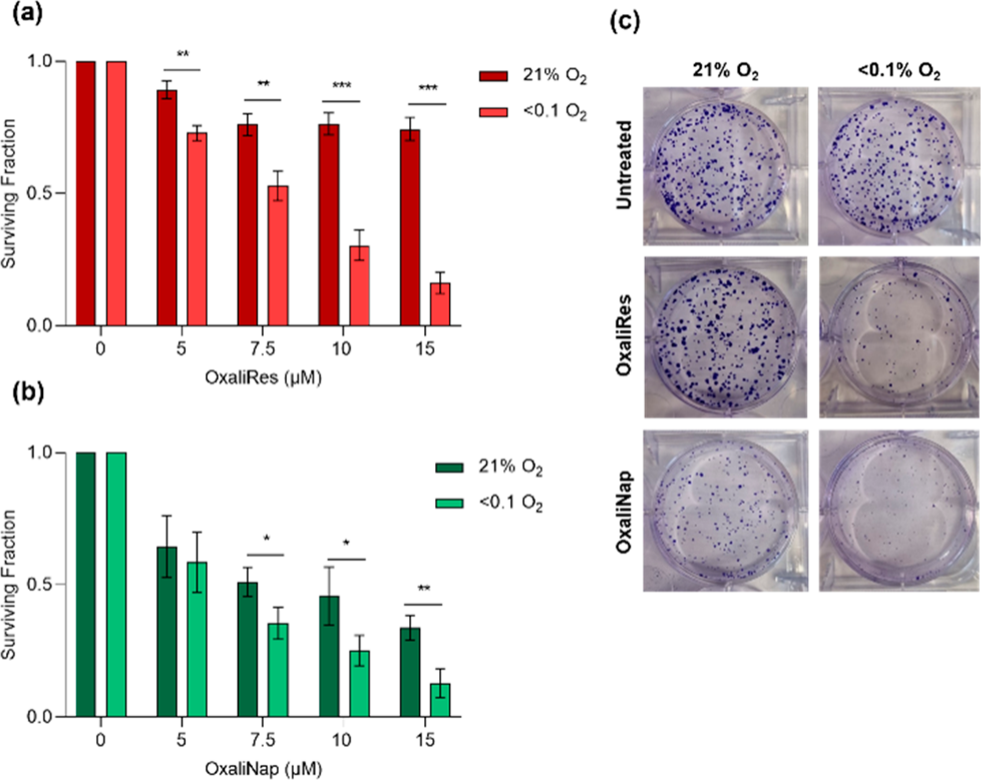
HCT116 cells were treated with the indicated concentrations of OxaliRes (a) or OxaliNap (b) for 3 days. Hypoxic cells were exposed to <0.1% O_2_ for 16 h. Cell survival was measured via clonogenic assay. (c) Representative images of clonogenic assay. Error bars represent SD * *p* < 0.05, ** *p* < 0.01, and *** *p* < 0.001. *n* = 3.

In summary, two fluorescence responsive OxPt(IV) complexes, OxaliRes and OxaliNap, were synthesized and the photophysical properties were evaluated in solution and in cancer cells. Solution studies showed both complexes responded to NaAsc, resulting in increases in their respective fluorescence emission intensities, 585 and 545 nm. Fluorescence imaging identified the reducing nature of tumor hypoxia was capable of inducing the bioreduction of OxPt(IV) to OxPt(II). At <0.1% O_2_, the greatest fluorescence signal was observed for both OxaliRes and OxaliNap, and a significant difference in cytotoxicity was observed when compared to normoxic conditions (21% O_2_). We thus believe this study has (1) identified carbamate modified OxPt(IV) complexes as potential hypoxia-activated prodrugs and (2) highlighted the importance of using imaging alongside drug development.

## Supporting Information Available

Full experimental procedures and characterization: ^1^H NMR, ^13^C NMR, LC–MS, and HRMS; additional figures; and UV spectra (PDF)
