# Supplementary material for: Oxali(IV)Fluors: Fluorescence Responsive Oxaliplatin(IV) Complexes Identify a Hypoxia-Dependent Reduction in Cancer Cells
Source: J Am Chem Soc. 2023 Jun 7;145(24):12998–3002. doi: 10.1021/jacs.3c03320 (PMC10288503; doi:10.1021/jacs.3c03320)
Supplement: Supplementary file 1 — ja3c03320_si_001.pdf [file ja3c03320_si_001.pdf]

Supporting Information

Oxali(IV)Fluors: Fluorescence Responsive Oxaliplatin(IV) Complexes Identify a Hypoxia-dependent Reduction in Cancer Cells

Marie H. C. Boulet,[a‡] Hannah R. Bolland,[b‡] Ester M. Hammond*,[b] and Adam C. Sedgwick*[a]

[a]Chemistry Research Laboratory, University of Oxford, Mansfield Road, OX1 3TA, United Kingdom

[b]Department of Oncology, University of Oxford, Old Road Campus Research Building, Oxford, OX3 7DQ, United Kingdom

Emails: ester.hammond@oncology.ox.ac.uk and adam.sedgwick@chem.ox.ac.uk

Table of Contents

1. General Information and Methods.................................. S3
2. Synthetic Schemes ……………………………………………………………S5
3. LC traces………………………………………………………………………S7
4. Additional Analyses…………………………………………………………..S8
5. Synthetic procedures S27
6. NMR Spectra.. S34
7. References.. S49

1. General Information and Methods

1.1 Materials and Chemicals

All chemicals and reagents were purchased commercially and were of analytical grade. Absorption spectra were obtained on a Jasco V-770 spectrophotometer and fluorescence spectra were obtained on a PerkinElmer LS55 Luminescence spectrometer using quartz cuvettes of 1 cm path length. Column chromatography was carried out using Merck® silica gel 60 under a positive pressure of nitrogen. Eluent ratios are reported by volume percentages. NMR spectra were recorded on a Bruker AVIII 400, Bruker NEO 600, Bruker AVII 500 (with cryoprobe) and Bruker AVIII 500 spectrometers. Chemical shifts are reported as δ values in ppm. Mass spectra were performed using Waters Micromass LCT and Bruker microTOF spectrometers. HPLC analysis: Agilent 1260 Infinity II® Poroshell 120 EC-C18 column [4 µm, 4.6 × 100 mm]; [95:5 H_2O: MeCN → 5:95 H_2O: MeCN: H_2O with 0.1% FA modifier, 10 min; 5 min hold; 1 mL min^{-1}]. LCMS Analysis: Agilent 1260 Infinity II® ZORBAX SB C18 column [1.8 µm, 2.1 × 50 mm]; [95:5 H_2O: MeCN → 5:95 H_2O: MeCN: H_2O with 0.1% FA modifier, 10 min; 5 min hold; 0.4 mL min^{-1}]. Cyclic voltammetry was performed in a Mbraun UNILab glovebox with a PalmSens Emstat3+ Blue potentiostat using a glassy carbon working electrode, a Pt wire as counter electrode and an Ag wire as pseudo-reference electrode.

1.2 Cell Experiments

1.2.1 Cell Culture

FLO-1 (Prof. Ricky Sharma, UCL), and HCT116 (Prof. Bert Vogelstein, Johns Hopkins Medicine) were cultured at 37°C, 5% CO_2 in a humidified incubator. Cells were grown in DMEM medium supplemented with 10% FBS. All cell lines were routinely mycoplasma tested using a HEK-Blue™ detection kit (Invivogen) and found to be negative. Inhibitors/compounds used were Ascorbate (Sigma Aldrich).

1.2.2 Hypoxia exposure

Hypoxia treatments at <0.1% O_2 were carried out in a Bactron II Chamber (Shel Laboratory). Oxygen concentrations were periodically validated using anaerobic oxygen indicator strips (ThermoFisher). Hypoxia treatments at 0.5-4% O_2 were carried out in a M35 variable atmosphere workstation (Don Whitley Scientific).

1.2.3 Flow Cytometry

Cells were seeded onto glass 6 cm^2 dishes. Cells were treated with 10 µM of probe. Cells were scraped in 1 mL of 1x PBS into a 1.5 mL tube and fixed inside the hypoxia chamber with 4% PFA for 10 minutes. Samples were washed three times with 1 x PBS. Samples were run on a CytoFLEX (Beckman Coulter) and data analyzed using FlowJo software. Filter sets used for OxaliNap were FITC (ex 488 nm / em 525/40 nm) and for OxaliRes PC5.5 (ex 561 nm / em 690/50 nm).

1.2.4 Fluorescent Microscopy

Cells were seeded onto autoclaved cover slips (Menzel-Glaser) before treatment. Cells were fixed inside the hypoxia chamber in 4% PFA (*w/v* paraformaldehyde in PBS) for 10 minutes. Cells were mounted onto microscopy slides (Menzel Glaser) with ProLong™ Gold Antifade Mountant with DAPI to visual the nucleus (Invitrogen™). Cells were visualized with an LSM780 confocal microscope (Carl Zeiss Microscopy Ltd) at 63x magnification. At least 100 cells were counted per condition for quantification. Excitation and emission wavelengths used were: DAPI 370/470 nm, OxaliRes 571/584 nm and OxaliNap 491/516 nm.

1.2.5 Colony Survival Assay

Cells were seeded at low density in 6-well plates and incubated for 4 h at 37°C to adhere. Cells were incubated at indicated oxygen concentrations for 16 h. Colonies were returned to normoxic conditions, underwent a media change after 3 days and then left to form colonies for 7-10 days. Colonies were stained with 2% crystal violet diluted in 50% methanol and 20% ethanol and counted manually. Plating efficiency was calculated by dividing the number of colonies by the number of cells seeded. Surviving fraction was determined by dividing plating efficiency for treatment by the plating efficiency for the respective control.

1.2.6 MTT Assay

4,000 RKO cells per well were seeded in 96 well plates and allowed to adhere overnight. Cells were treated with the indicated probe and concentration for 3 days. Cells were exposed to hypoxia (<0.1% O_2) for 16 hours. This was the same time point used for the clonogenic assays performed in HCT116 cells. Cells were incubated with 0.5 mg/mL MTT reagent (Sigma) in complete media for 3 hours at 37 °C protected from light. MTT was removed and formazan crystals were solubilized with 100 µL of DMSO for 15 minutes at 37 °C protected from light. Absorbance was read at 570 nm (Clariostar, BMG). Data are shown as percentage viability relative to untreated control.

2. Synthetic Schemes

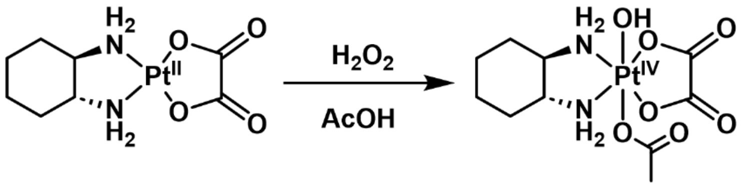

1

Scheme S1. Synthesis of **OxPt(OH)(OAc)** (**1**)

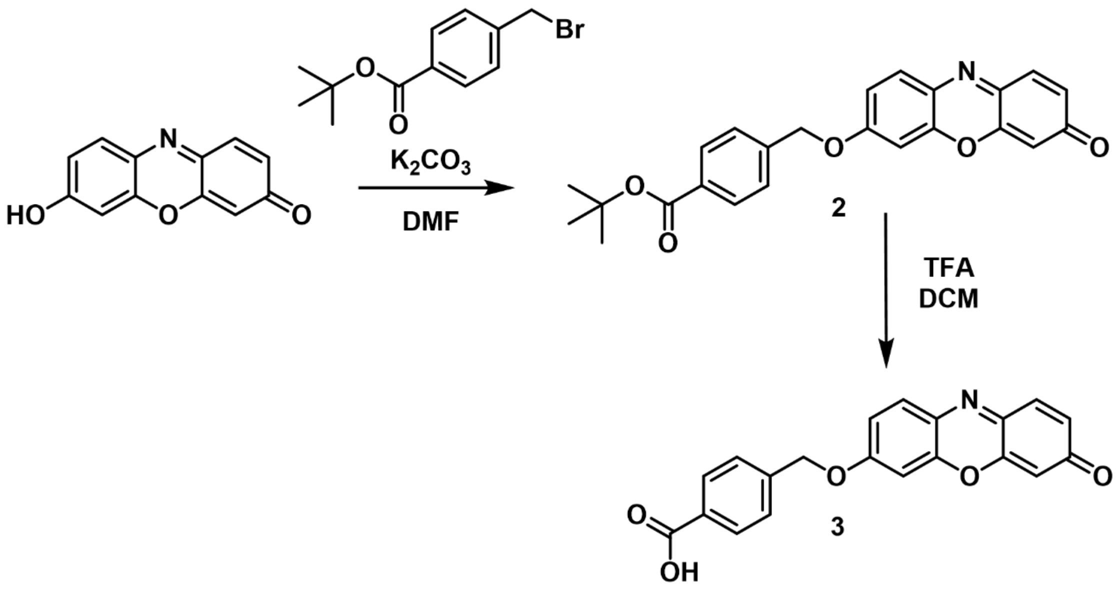

2

3

Scheme S2. Synthesis of **4-(((3-Oxo-3*H*-phenoxazin-7-yl)oxy)methyl)benzoic acid** (**3**)

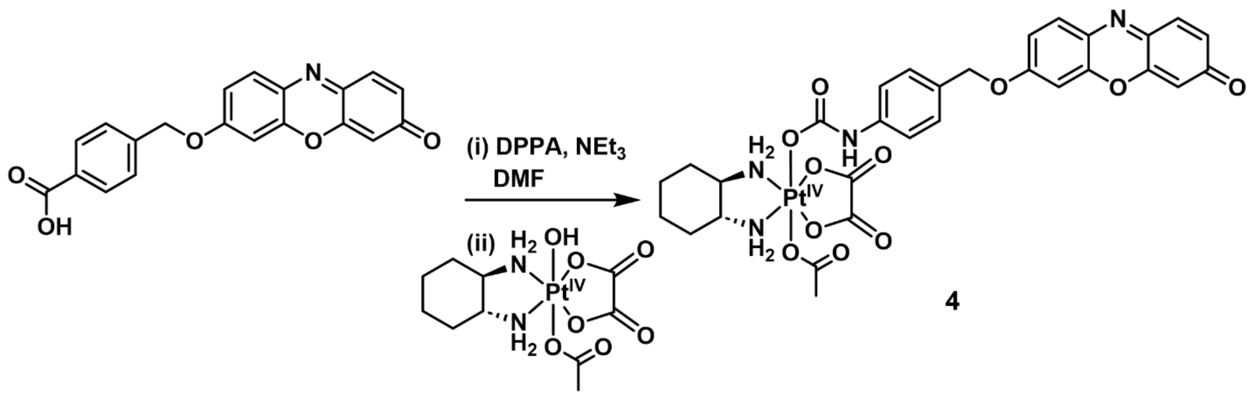

Scheme S3. Synthesis of **OxaliRes** (**4**)

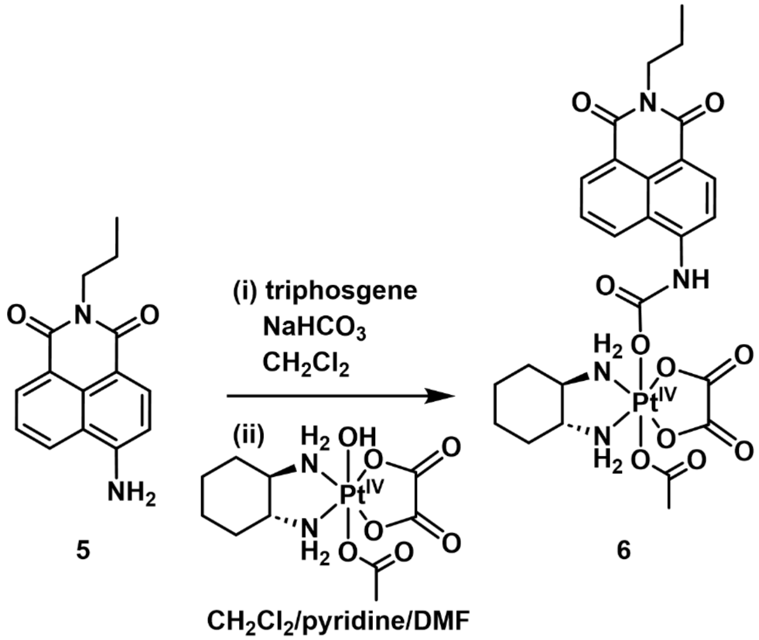

Scheme S4. Synthesis of **OxaliNap** (**6**)

3. LC traces

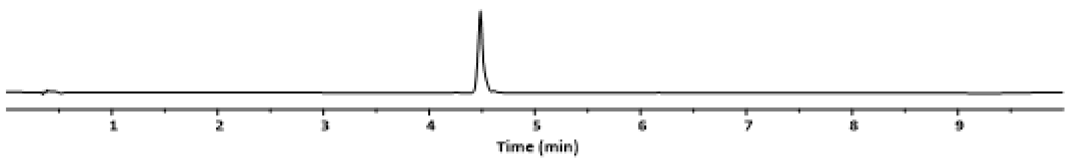

Figure S1. LC trace of **OxaliRes** (**4**) (Absorption at 300 nm)

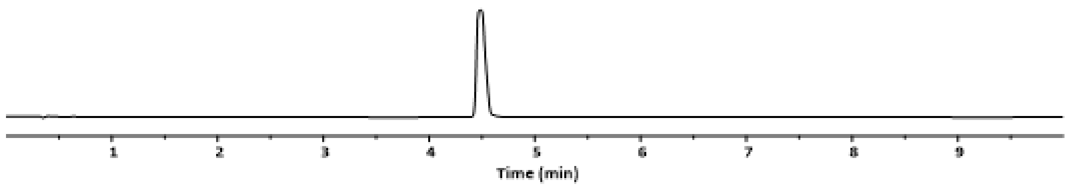

Figure S2. LC trace of **OxaliNap** (**6**) (Absorption at 360 nm).

4. Additional Analyses

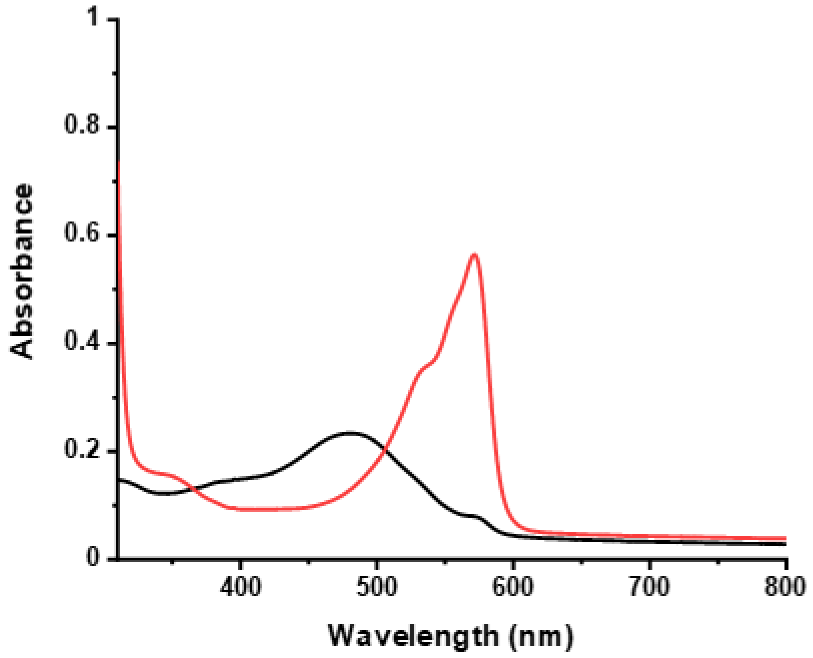

Figure S3. Absorbance spectra of **OxaliRes** (15 µM) in PBS buffer (pH = 7.40) before (**black**) and after (**red**) addition of NaAsc (4 mM). The second measurement was recorded after 60 minutes incubation.

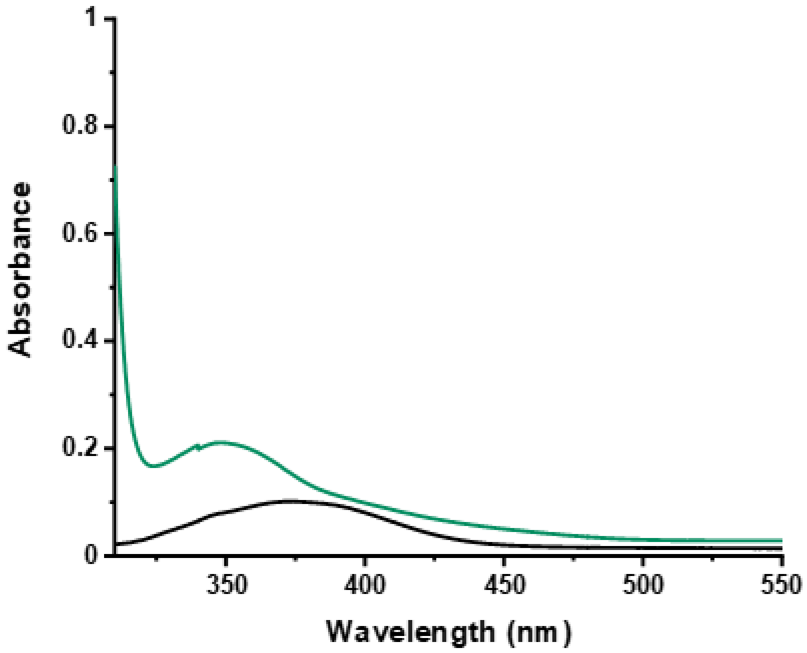

Figure S4. Absorption spectra of **OxaliNap** (15 µM) in PBS buffer (pH = 7.40) before (**black**) and after (**green**) addition of NaAsc (4 mM). The second measurement was recorded after 60 minutes incubation.

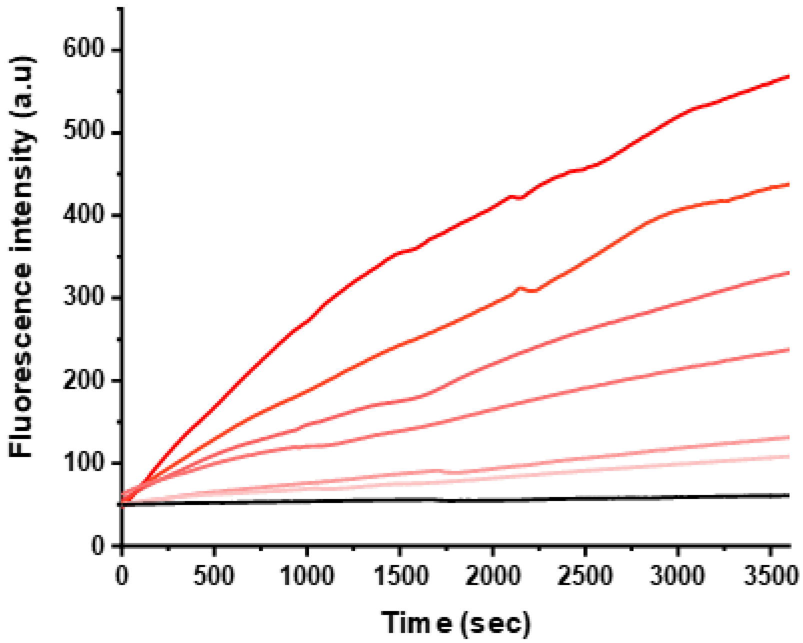

Figure S5. Changes in fluorescence emission intensity of **OxaliRes** (5 µM) at 585 nm with different concentrations of NaAsc (125 µM, 250 µM, 500 µM, 1 mM, 2 mM, 4 mM) over one hour. All measurements were performed in PBS buffer (pH = 7.40), λ_{ex} = 500 nm (Slit widths: 10 nm and 2.5 nm).

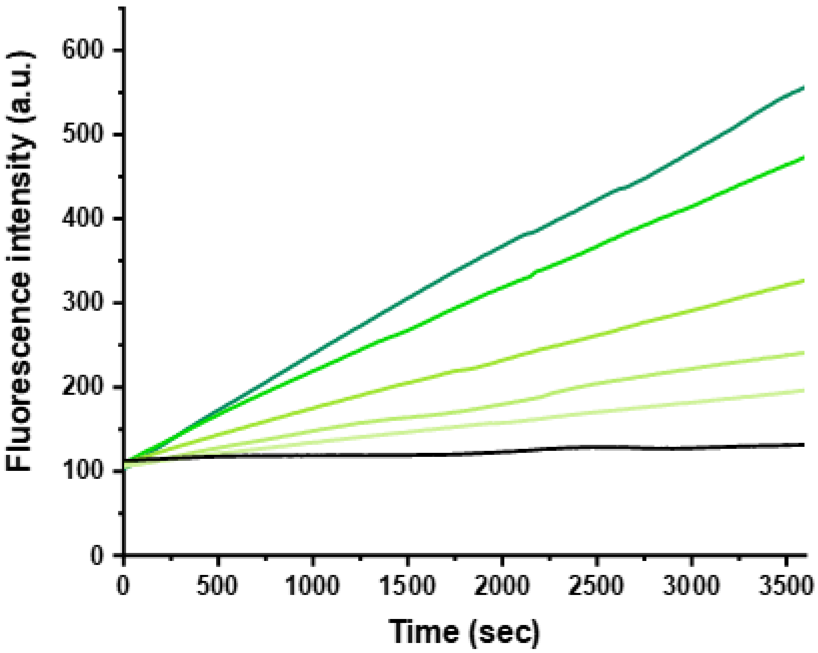

Figure S6. Changes in fluorescence emission intensity of **OxaliNap** (5 µM) at 545 nm with different concentrations of NaAsc (1 mM, 2 mM, 4 mM, 8 mM, 10 mM) over one hour. All measurements were performed in PBS buffer (pH = 7.40), λ_{ex} = 430 nm (Slit widths: 10 nm and 10 nm).

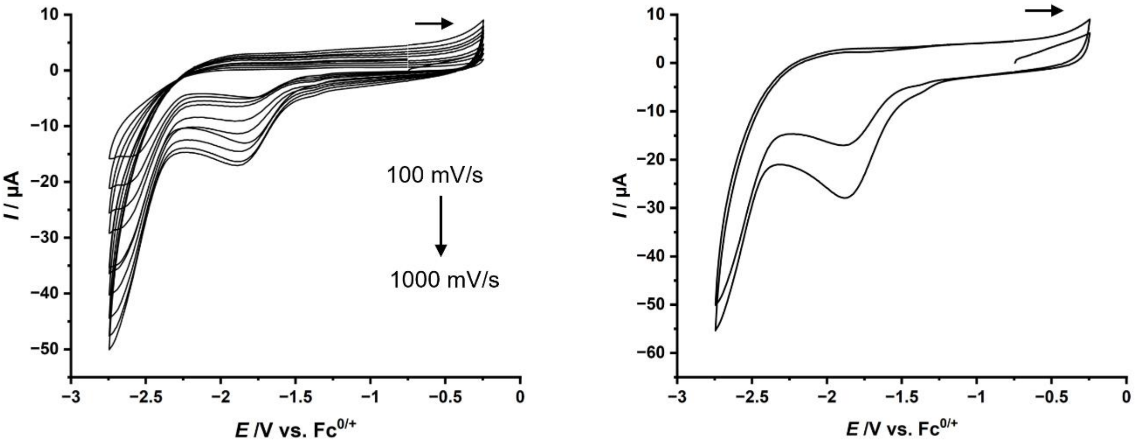

Figure S7. Left: Cyclic voltammogram (DMF, 0.1 M $[Bu_4N][PF_6]$) of a 1 mM solution of OxPt(OH)(OAc) (**1**). Potentials are referenced to $[Cp_2Fe]^{+/0}$. Right: Cyclic voltammogram (DMF, 0.1 M $[Bu_4N][PF_6]$) of a 1 mM solution of OxPt(OH)(OAc) (**1**). Potentials are referenced to $[Cp_2Fe]^{+/0}$. The scan rate was 1000 mV/S.

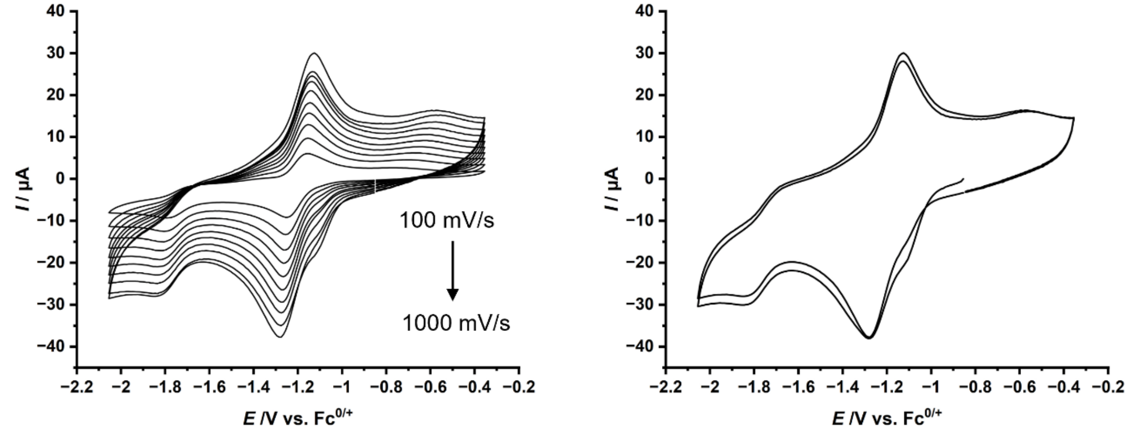

Figure S8. Left: Cyclic voltammogram (DMF, 0.1 M $[Bu_4N][PF_6]$) of a 1 mM solution of OxaliRes (**4**). Potentials are referenced to $[Cp_2Fe]^{+/0}$. Right: Cyclic voltammogram (DMF, 0.1 M $[Bu_4N][PF_6]$) of a 1 mM solution of OxaliRes (**4**). Potentials are referenced to $[Cp_2Fe]^{+/0}$. The scan rate was 1000 mV/S.

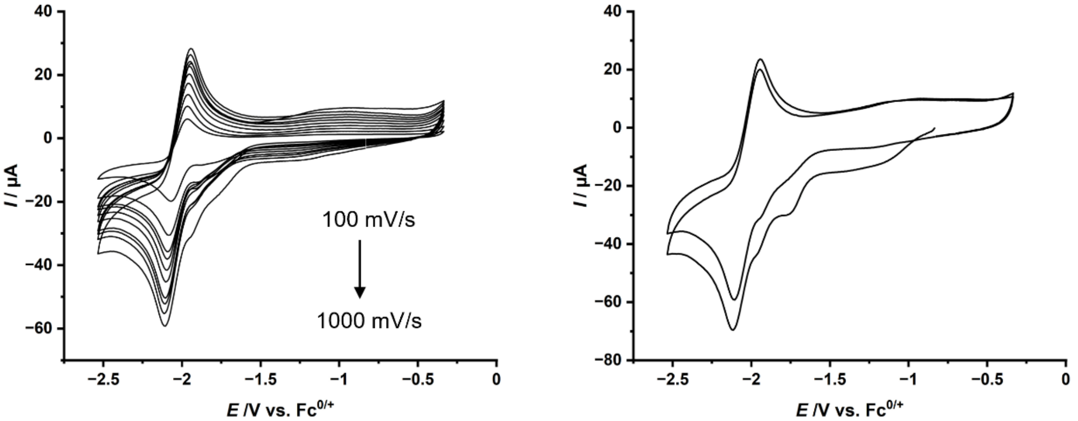

Figure S9. Left: Cyclic voltammogram (DMF, 0.1 M $[Bu_4N][PF_6]$) of a 1 mM solution of OxaliNap (**6**). Potentials are referenced to $[Cp_2Fe]^{+/0}$. Right: Cyclic voltammogram (DMF, 0.1 M $[Bu_4N][PF_6]$) of a 1 mM solution of OxaliNap (**6**). Potentials are referenced to $[Cp_2Fe]^{+/0}$. The scan rate was 1000 mV/S.

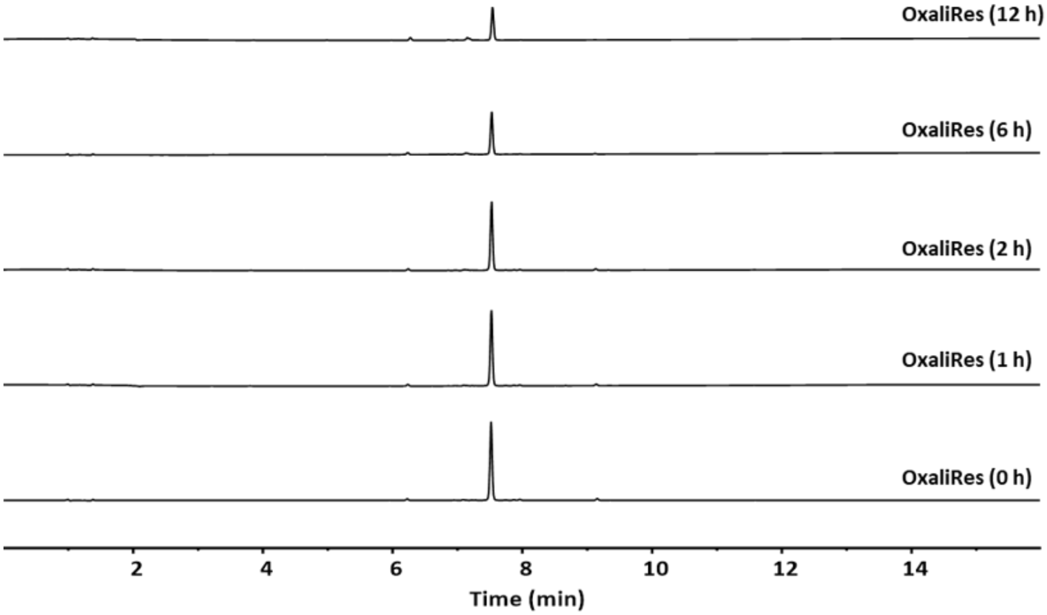

Figure S10. Stability study of **OxaliRes** (**4**) in PBS buffer (pH 7.40) recorded by HPLC (Absorption at 254 nm).

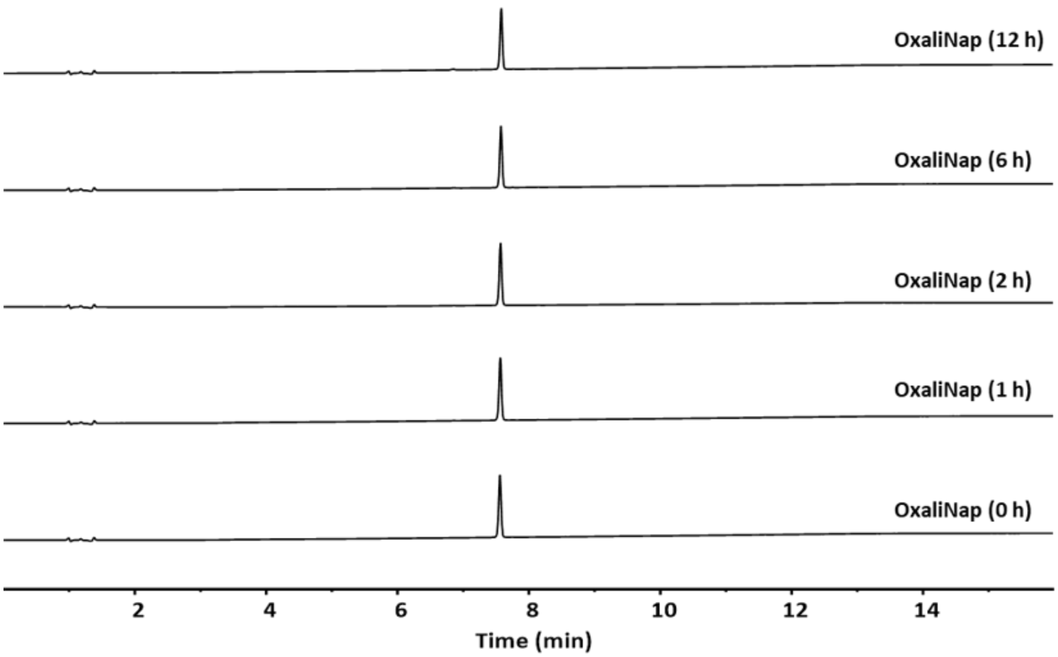

Figure S11. Stability study of **OxaliNap** (**6**) in PBS buffer (pH 7.40) recorded by HPLC (Absorption at 360 nm).

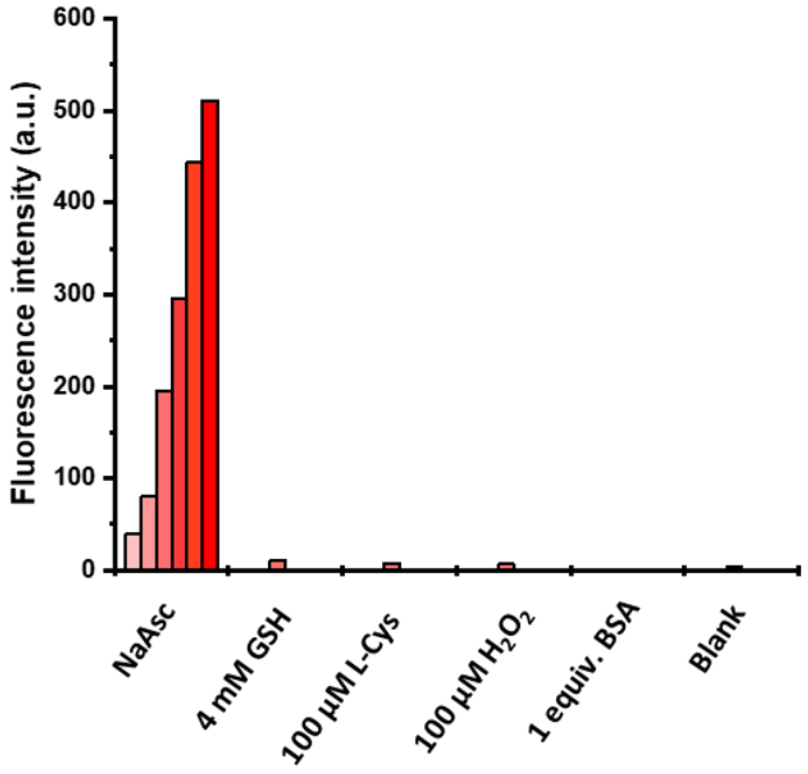

Figure S12. Relative fluorescence intensity of **OxaliRes** (5 µM) in the presence of various analytes: NaAsc (125 µM, 250 µM, 500 µM, 1 mM, 2 mM, 4 mM); GSH (4 mM); L-Cysteine (100 µM); H_2O_2 (100 µM) and BSA (1 equiv.).

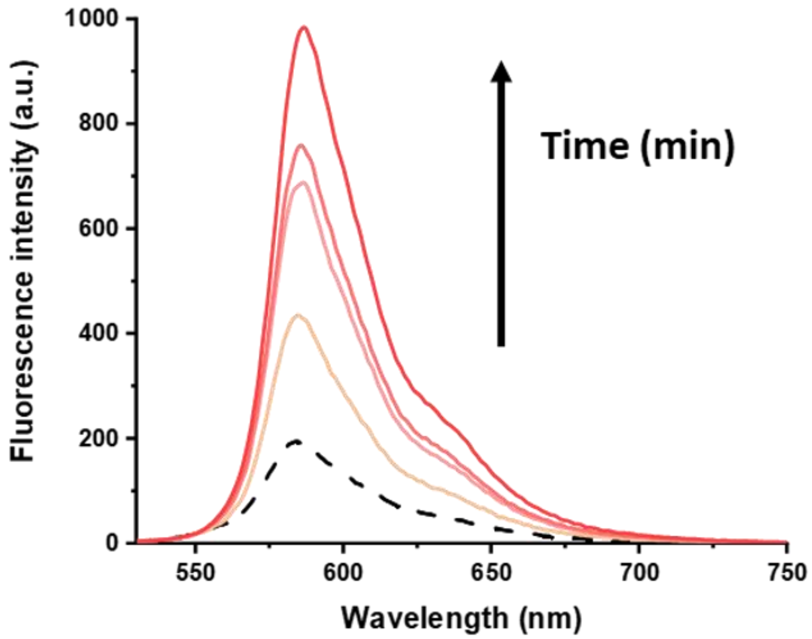

Figure S13. Changes in fluorescence emission intensity of **OxaliRes** (5 μM) in the presence of NADH (0.5 mM) at different time points (0 min, 5 min, 10 min, 15 min, 20 min). All measurements were performed in PBS buffer (pH = 7.40), λ_{ex} = 500 nm (Slit widths: 10 nm and 2.5 nm).

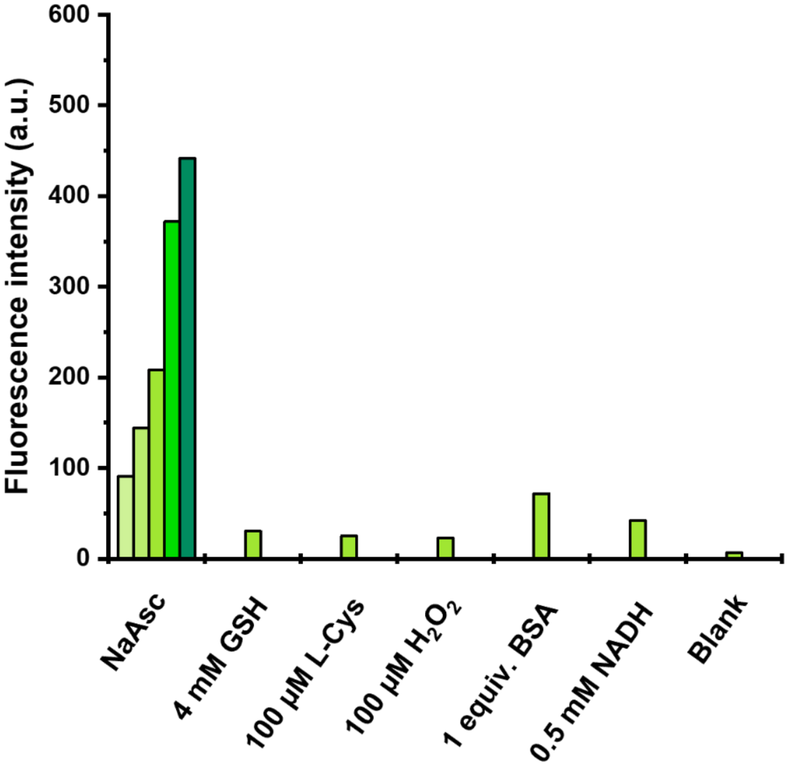

Figure S14. Relative fluorescence intensity of **OxaliNap** (5 µM) in the presence of various analytes: NaAsc (1 mM, 2 mM, 4 mM, 8 mM, 10 mM); GSH (4 mM); L-Cysteine (100 µM); H_2O_2 (100 µM); BSA (1 equiv.) and NADH (0.5 mM).

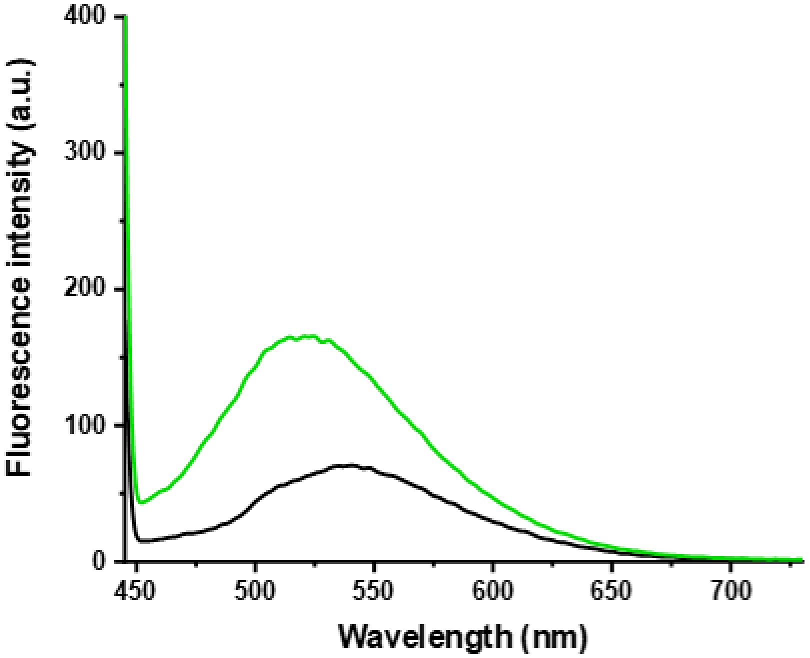

Figure S15. Relative fluorescence intensity of **OxaliNap** (5 µM) in PBS buffer (pH = 7.40) before (**black**) and after (**green**) addition of BSA (1 equiv.). The second measurement was recorded after 60 minutes incubation.

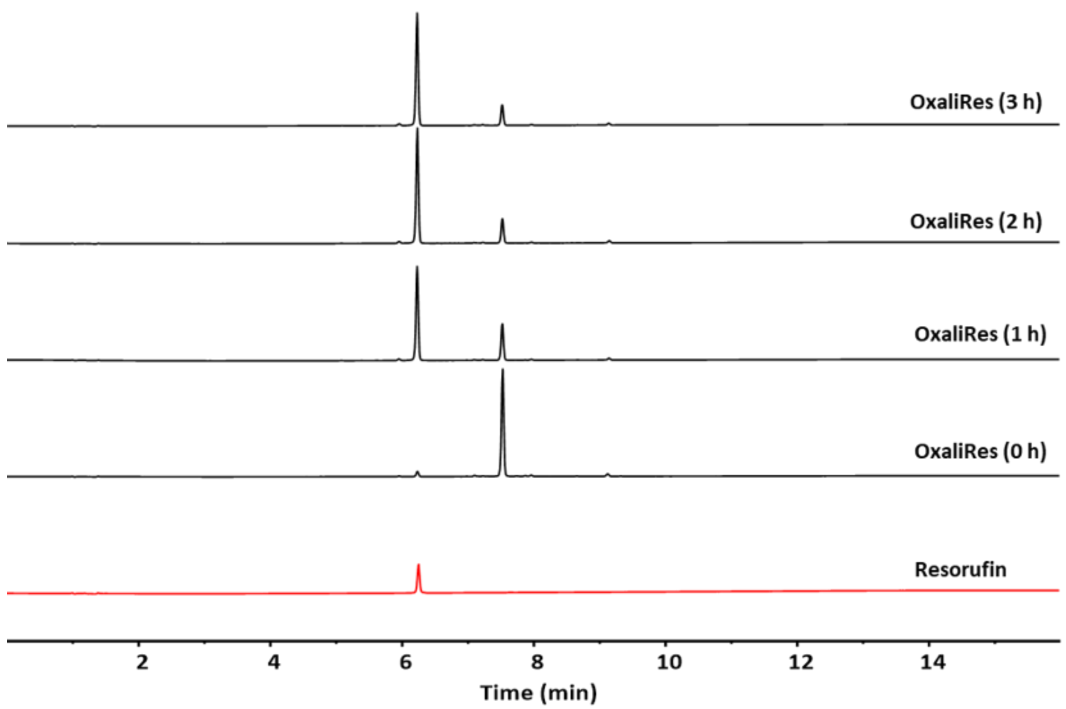

Figure S16. The reduction of **OxaliRes** (**4**) in PBS buffer (pH 7.40) with 1 mM sodium ascorbate recorded by HPLC (Absorption at 480 nm).

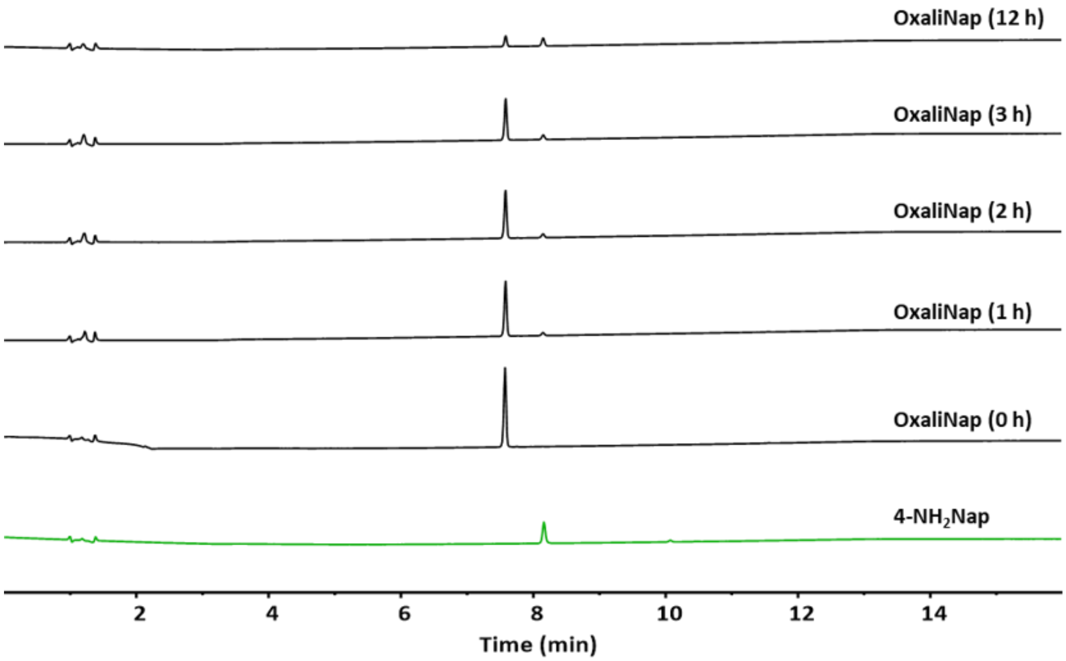

Figure S17. The reduction of **OxaliNap** (**6**) in PBS buffer (pH 7.40) with 8 mM sodium ascorbate recorded by HPLC (Absorption at 360 nm).

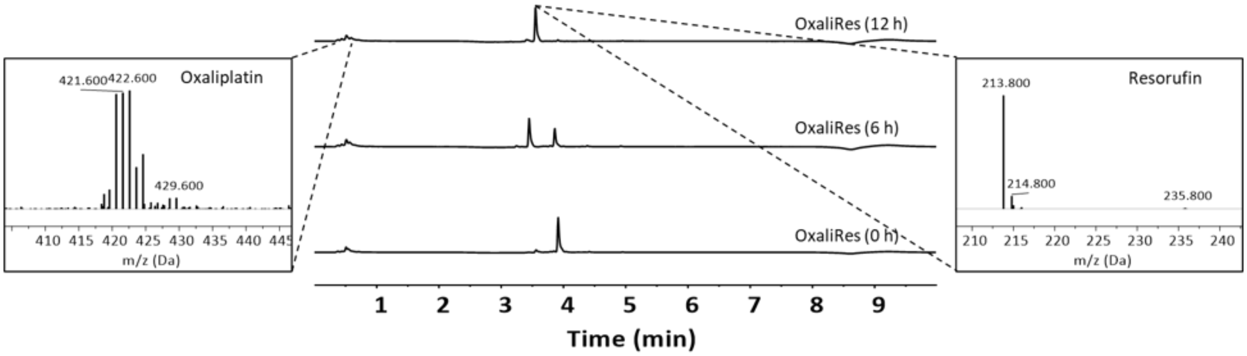

Figure S18. The reduction of **OxaliRes** (**3**) in deionised H_2O containing 1 mM sodium ascorbate over 12 h. The reduction process and products were recorded by LC-MS. Oxaliplatin was observed as the Na+ adduct.

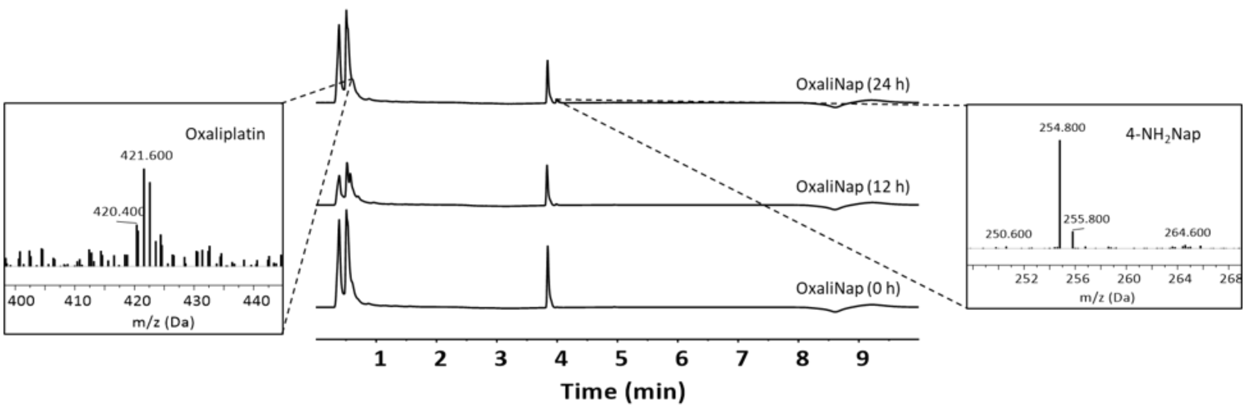

Figure S19. The reduction of **OxaliNap** (**6**) in deionised H_2O containing 4 mM sodium ascorbate over 24 h. The reduction process and products were recorded by LC-MS. Oxaliplatin was observed as the Na+ adduct.

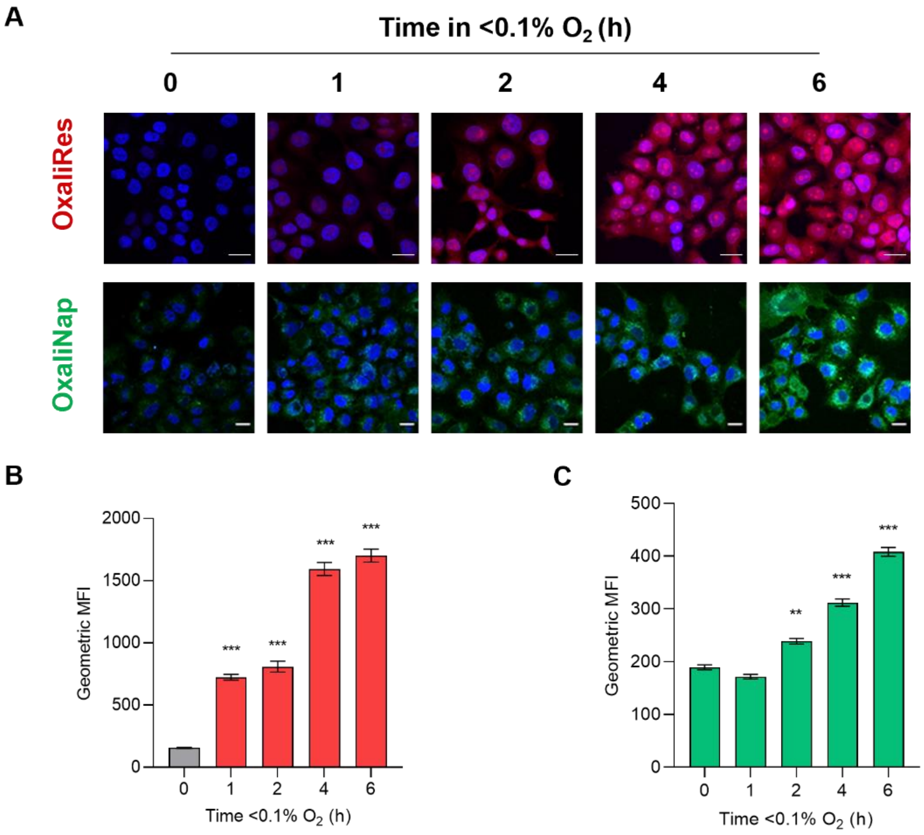

Figure S20. HCT116 cells were treated with **OxaliRes** or **OxaliNap** (10 µM) for the times indicated (0-6 h) in <0.1% O_2. **A**. Representative images are shown. Images were taken at 63x magnification. Scale bar represents 20 µM. HCT116 cells were treated with **OxaliRes** (10 µM) for the times indicated in hypoxia followed by flow cytometry. HCT116 cells were treated with **OxaliNap** (10 µM) for the times indicated in hypoxia followed by flow cytometry. In **B** and **C** relative fluorescence is displayed as geometric mean intensity (MFI). Error bars represent SD. Significance compared to normoxic control. * $p < 0.05$, ** $p < 0.01$, and *** $p < 0.001$. n=3.

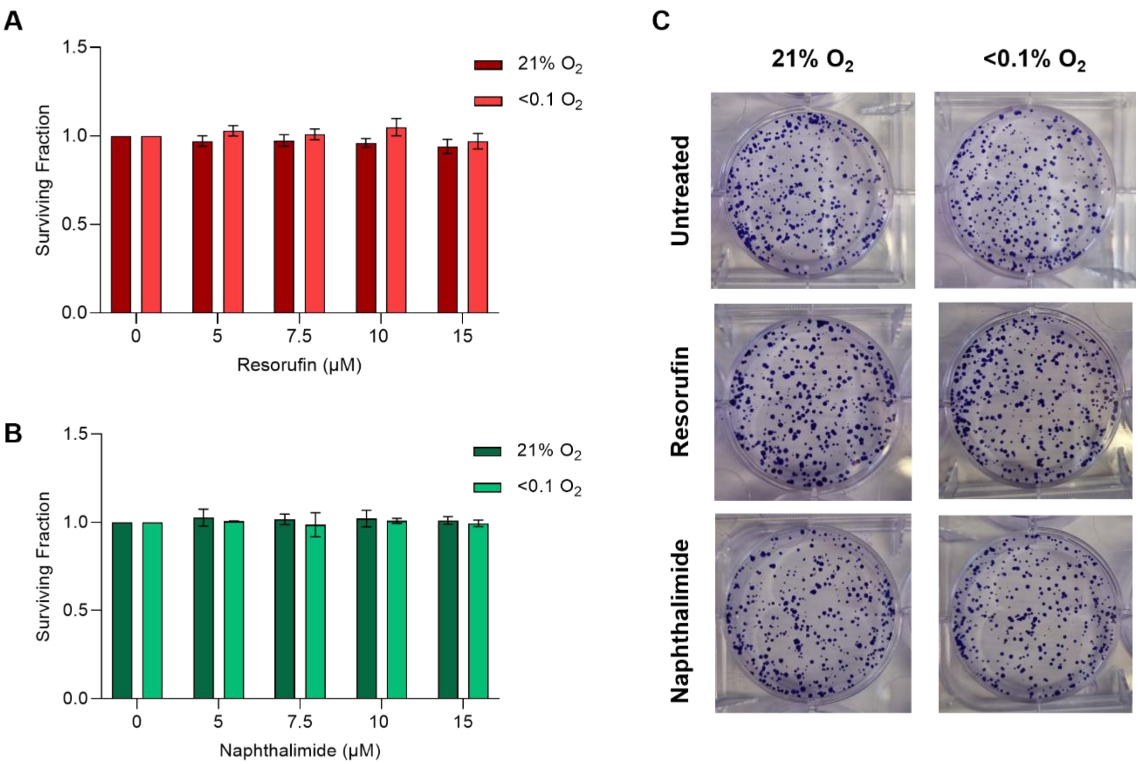

Figure S21. HCT116 cells were treated with the indicated concentrations of Resorufin or Naphthalimide (4-NH_2Nap) for 3 days. Hypoxic cells were exposed to <0.1% O_2 for 16 hours with addition of either Resorufin or Naphthalimide. Cell survival was measured via clonogenic assay. **A**. Resorufin surviving fraction. **B**. Naphthalimide surviving fraction. **C**. Representative images of clonogenic assay. Error bars represent SD.

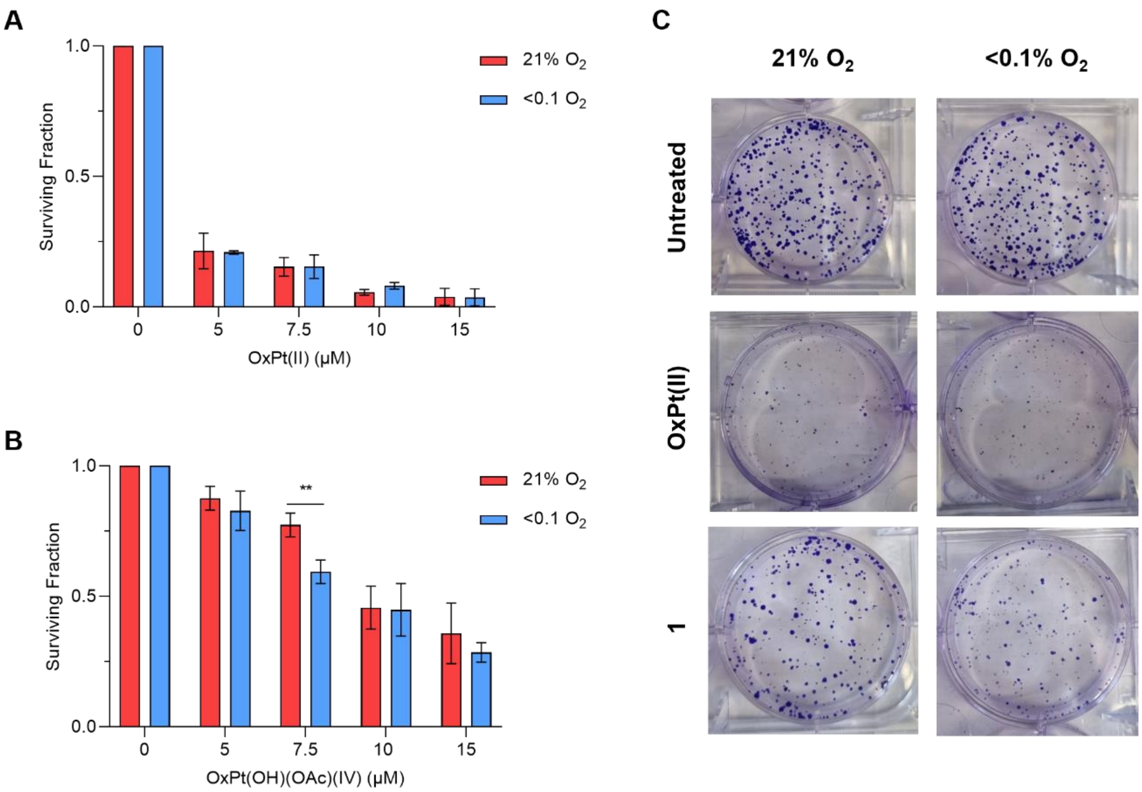

Figure S22. HCT116 cells were treated with the indicated concentrations of Oxaliplatin(OxPt(II)) or OxPt(OH)(OAc)(IV) (**1**) for 3 days. Hypoxic cells were exposed to <0.1% O_2 for 16 hours with addition of either OxPt(II) or **1**. Cell survival was measured via clonogenic assay. **A**. OxPt(II) surviving fraction. **B**. **1** surviving fraction. **C**. Representative images of clonogenic assay. Error bars represent SD. * p < 0.05, ** p < 0.01, and *** p < 0.001. n=3.

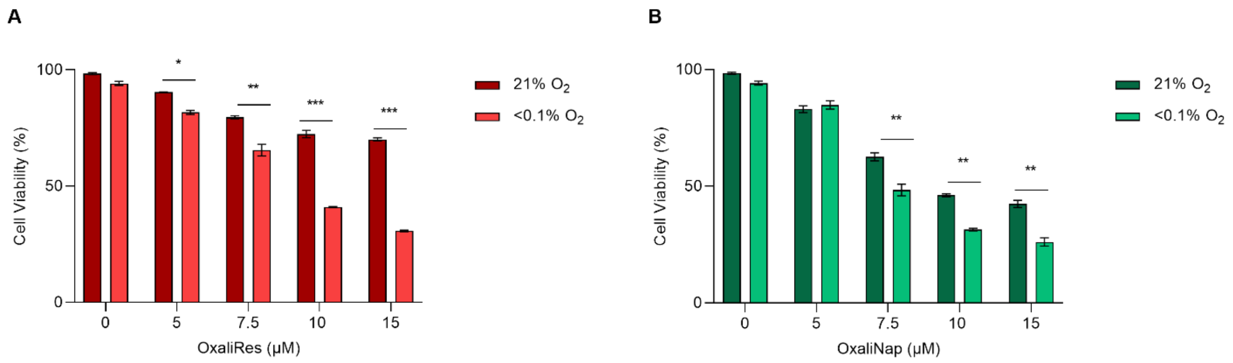

Figure S23. RKO cells were treated with the indicated concentrations of OxaliRes or OxaliNap for 3 days. Hypoxic cells were exposed to <0.1% O_2 for 16 hours with addition of either OxaliRes or OxaliNap. Cell viability was measured via MTT assay. **A**. OxaliRes cell viability. **B**. OxaliNap cell viability. Data presented are percentage cell viability relative to untreated control. Error bars represent SD. * $p < 0.05$, ** $p < 0.01$, and *** $p < 0.001$. n=3.

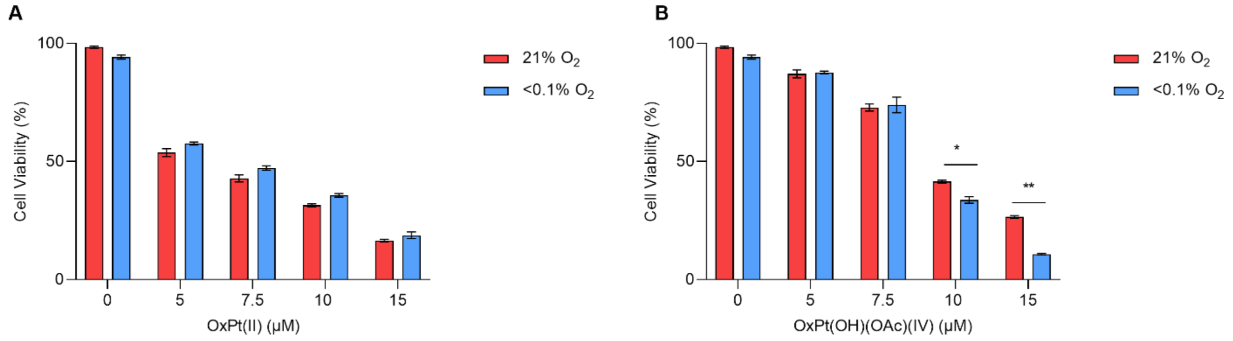

Figure S24. RKO cells were treated with the indicated concentrations of OxPt(II) or **1** for 3 days. Hypoxic cells were exposed to <0.1% O_2 for 16 hours with addition of either OxPt(II) or **1**. Cell viability was measured via MTT assay. **A**. OxPt(II) cell viability. **B**. **1** cell viability. Data presented are percentage cell viability relative to untreated control. Error bars represent SD. * $p < 0.05$, ** $p < 0.01$, and *** $p < 0.001$. n=3.

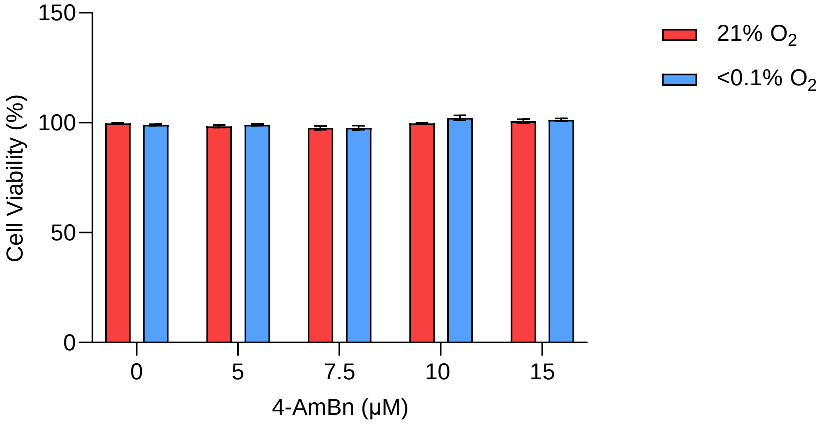

Figure S25. RKO cells were treated with the indicated concentrations of 4-AmBn for 3 days. Hypoxic cells were exposed to <0.1% O_2 for 16 hours with addition of 4-AmBn. Cell viability was measured via MTT assay. Data presented are percentage cell viability relative to untreated control. Error bars represent SD. * $p < 0.05$, ** $p < 0.01$, and *** $p < 0.001$. n=3.

5. Synthetic Procedures

OxPt(OH)(OAc) (1)

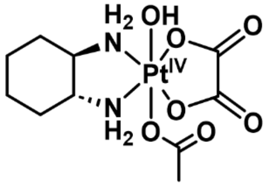

The title compound was synthesized following a previously reported literature procedure.[1] In brief, hydrogen peroxide – 34.5-36.5 % in water (2 mL) was added to oxaliplatin (0.200 g, 0.503 mmol) in acetic acid (80 mL) and the reaction was stirred overnight at room temperature. The solvent was removed under reduced pressure and the residual solid was washed with diethyl ether to obtain a white solid (Quant. Yield). ^{1}H NMR (500 MHz, D_2O) δ_H: 2.87 (m, 2H), 2.30 (m, 2H), 2.06 (s, 3H), 1.65-1.55 (m, 4H), 1.25 (m, 2H) ppm. ^{195}Pt NMR (107 MHz, D_2O) δ: 1315 ppm.

***tert*-Butyl 4-(((3-oxo-3*H*-phenoxazin-7-yl)oxy)methyl)benzoate (2)**

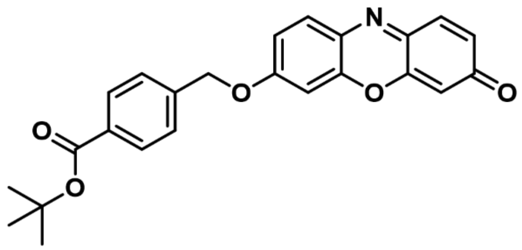

Resorufin (0.500 g, 2.35 mmol) was added to *tert*-butyl 4-(bromomethyl)benzoate (0.637 g, 2.35 mmol) and K_2CO_3 (0.487 g, 3.525 mmol) in DMF (3 mL). The reaction mixture was stirred at room temperature overnight. The reaction was diluted with CH_2Cl_2 (30 mL) and brine (30 mL) and the organic layer was washed with brine (4 x 50 mL), dried with $MgSO_4$ and concentrated under reduced pressure to afford the title compound as a bright red solid (0.535 g, 1.33 mmol, 56 %). ^{1}H NMR (400 MHz, $CDCl_3$) δ 8.04 (d, *J* = 8.4 Hz, 2H), 7.75 (d, *J* = 8.8 Hz, 1H), 7.49 (d, *J* = 9.4 Hz, 2H), 7.44 (s, 1H), 7.05 (dd, *J* = 2.6, 9.0 Hz, 1H), 6.91 (d, *J* = 2.0 Hz, 1H), 6.88 (bs, 1H), 6.43 (d, *J* = 2.0 Hz, 1H), 5.25 (s, 2H), 1.59 (s, 9H) ppm. ^{13}C NMR (151 MHz, $CDCl_3$) δ 186.1, 165.3, 162.7, 149.9, 145.8, 145.7, 139.9, 134.9, 134.1, 132.3, 131.9, 130.1, 128.9, 127.0, 114.6, 106.8, 101.3, 81.4, 70.4, 28.3 ppm. HRMS: *m/z* calculated for $C_{24}H_{21}NO_5$, $[M+H]^+$ 404.1493, found 404.1489.

4-(((3-Oxo-3*H*-phenoxazin-7-yl)oxy)methyl)benzoic acid (3)

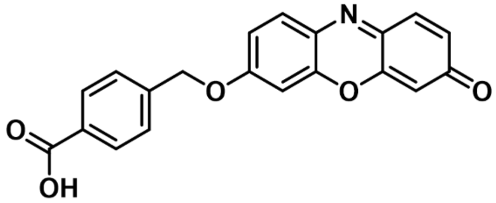

Tert-butyl 4-(((3-oxo-3*H*-phenoxazin-7-yl)oxy)methyl)benzoate (0.515 g, 1.28 mmol) was dissolved in CH_2Cl_2 (5 mL). Trifluoroacetic acid (5 mL) was added and the reaction mixture was stirred at room temperature for 3 hours. The solvent was removed under reduced pressure, washed twice with MeOH and dried to give a bright red solid (0.435 g, 1.25 mmol, 98 %). ^{1}H NMR (400 MHz, DMSO-d$_6$) δ 13.02 (s, 1H), 7.99 (d, *J* = 8.0 Hz, 2H), 7.79 (d, *J* = 8.8 Hz, 1H), 7.60 (d, *J* = 8.0 Hz, 2H), 7.53 (d, *J* = 9.5 Hz, 1H), 7.19 (d, *J* = 2.4 Hz, 1H), 7.15 (dd, *J* = 2.4, 8.5 Hz, 1H), 6.79 (dd, *J* = 2.0, 9.6 Hz, 1H), 6.25 (d, *J* = 2.0 Hz, 1H), 5.37 (s, 2H) ppm. ^{13}C NMR (151 MHz, DMSO-d$_6$) δ 185.3, 167.0, 162.1, 157.8, 157.6, 149.7, 145.4, 145.2, 141.0, 134.9, 133.8, 131.4, 130.6, 129.6, 128.1, 127.6, 118.4, 116.4, 114.3, 105.7, 101.3, 69.7 ppm. HRMS: *m/z* calculated for $C_{20}H_{13}NO_5$ $[M+H]^+$ 348.0867, found 348.0862.

OxaliRes (4)

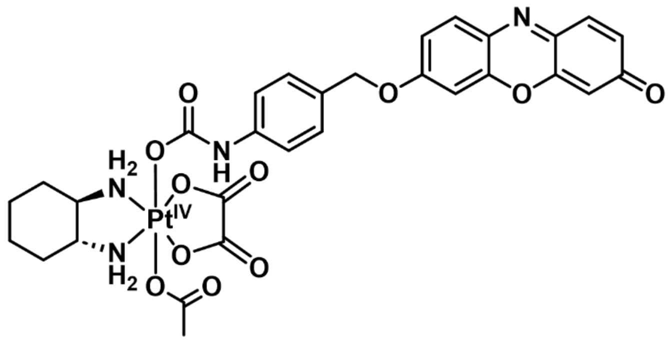

4-(((3-oxo-3*H*-phenoxazin-7-yl)oxy)methyl)benzoic acid (0.040 g, 0.107 mmol) was suspended in DMF (3 mL). EtN_3 (22 µL, 0.107 mmol) and diphenyl phosphoryl azide (DPPA) (34 µL, 0.107 mmol) were added and the reaction mixture was stirred for 30 min at room temperature followed by heating at 70 °C for 3 hours. OxPt(OH)(OAc) (0.024 g, 0.052 mmol) was added and the reaction was stirred at 100 °C. Upon completion, the crude mixture was purified via column chromatography (100 % Hexane to 100 % EtOAc to 5% MeOH in CH_2Cl_2) to afford the title compound as a red solid (0.015 g, 0.018 mmol, 51 %). ^{1}H NMR (600 MHz, DMSO-d$_6$) δ 9.21 (s, 1H), 8.50 (m, 4H), 7.77 (d, J = 8.8 Hz, 1H), 7.53 (d, J = 9.7 Hz, 1H), 7.45 (d, J = 7.8 Hz, 2H), 7.31 (d, J = 8.3 Hz, 2H), 7.17 (d, J = 2.7 Hz, 1H), 7.11 (dd, J = 2.6, 8.9 Hz, 1H), 6.79 (dd, J = 2.1, 9.8 Hz, 1H), 6.27 (d, J = 2.1 Hz, 1H), 5.17 (s, 2H), 2.75 (m, 1H), 2.64 (m, 1H), 2.16 (m, 2H), 1.97 (s, 3H), 1.52 (m, 2H), 1.47 (m, 2H), 1.19 (m, 2H) ppm. ^{13}C NMR (151 MHz, DMSO-d$_6$) δ 185.3, 178.2, 163.4, 163.4, 162.4, 161.0, 149.7, 145.2, 145.2, 140.1, 134.9, 133.7, 131.3, 128.9, 128.7, 127.9, 118.3, 114.4, 105.6, 101.2, 70.3, 61.1, 61.0, 30.9, 30.9, 30.8, 29.6, 23.5, 23.5, 22.9 ppm.^{195}Pt NMR (107 MHz, DMSO-d$_6$) δ 1626 ppm. HRMS: *m/z* calculated for $C_{30}H_{30}N_4O_{11}Pt$ $[M+Na]^+$ 840.1451, found 840.1449.

6-amino-2-propyl-1*H*-benzo[*de*]isoquinoline-1,3(2*H*)-dione (4-NH_2Nap; 5)

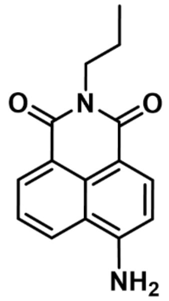

4-NH_2Nap (**5**) was synthesized following a previously reported literature procedure.[2] In brief, $SnCl_2$ (6.00 g, 32.00 mmol) was added to a solution of 4-NO_2Nap (1.50 g, 5.28 mmol) in MeOH (50 mL) and concentrated HCl (10 mL). The reaction mixture was stirred at room temperature and monitored by TLC. Upon completion, the solvent was removed under reduced pressure, and the aqueous layer was adjusted to a basic solution. The title compound was isolated via routine work up and silica column purification. ^{1}H NMR (400 MHz, DMSO-d$_6$) δ 8.60 (dd, 1H, *J* = 1.4, 8.5 Hz), 8.41 (dd, 1H, *J* = 1.4, 7.4 Hz), 8.18 (d, 1H, *J* = 8.5 Hz), 7.62 (t, 1H, *J* = 7.4 Hz), 7.41 (s, 2H), 6.84 (d, 1H, *J* = 8.3 Hz), 3.95 (t, 2H, *J* = 7.5 Hz), 1.60 (sext, 2H, *J* = 7.4 Hz), 0.89 (t, 3H, *J* = 7.4 Hz) ppm. ^{13}C NMR (151 MHz, DMSO-d$_6$) δ 163.7, 162.9, 152.6, 133.9, 130.9, 129.6, 129.2, 123.9, 121.8, 119.3, 108.1, 107.6, 40.7, 21.0, 11.4 ppm. HRMS: *m/z* calculated for $C_{15}H_{14}N_2O_2$ $[M+H]^+$ 255.1128, found 255.1123.

OxaliNap (6)

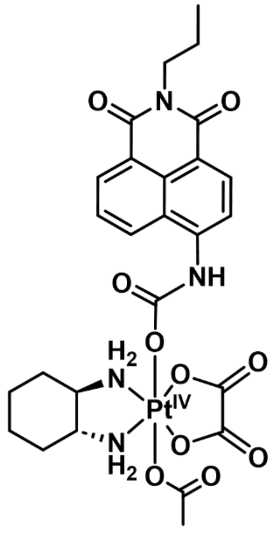

To a solution of 6-amino-2-propyl-1*H*-benzo[*de*]isoquinoline-1,3(2*H*)-dione (0.040 g, 0.157 mmol, 1.0 equiv.) in CH_2Cl_2 (3 mL) was added triphosgene (0.093 g, 0.314 mmol, 2.0 equiv.) and $NaHCO_3$ (0.040 g, 0.471 mmol, 3.0 equiv.). The mixture was stirred at room temperature for 3 hours. The crude product was added dropwise to a stirring solution of OxPt(OH)(OAc) (0.037 g, 0.079 mmol, 0.5 equiv.) in CH_2Cl_2 /pyridine /DMF (3:1:1) at 0 ^oC. After 5 minutes, the reaction mixture was warmed to room temperature and the solution was stirred for 1 hour. The solvent was removed under reduced pressure and the resulting yellow solid was redissolved in DMF and purified by column chromatography on silica gel (100 % Hexane to 100 % EtOAc to gradient method 2-4% MeOH in CH_2Cl_2) to give a yellow solid. The recovered solid was triturated with EtOAc and $CHCl_3$ to yield a yellow solid (0.020 g, 0.027 mmol, 34 %). ^1H NMR (600 MHz, DMSO-d_6) δ_H: 9.71 (s, 1H, NH), 8.66 (dd, *J* = 1.2, 8.4 Hz, 1H), 8.51 (dd, *J* = 1.2, 7.2 Hz, 1H), 8.44-8.30 (m, 4H), 8.42 (d, *J* = 8.3 Hz, 1H), 8.11 (d, *J* = 8.3 Hz,1H), 7.83 (t, *J* = 7.34 Hz, 1H), 4.00 (t, *J* = 7.4 Hz, 2H), 2.86 (m, 1H), 2.60 (m, 1H), 2.16-2.12 (m, 2H), 1.99 (s, 3H), 1.65 (sext, *J* = 8.4 Hz, 2H), 1.54-1.45 (m, 4H), 1.23-1.14 (m, 2H), 0.91 (t, *J* = 7.8 Hz, 3H) ppm. ^{13}C NMR (151 MHz, DMSO-d_6) δ_C: 178.3, 163.6, 163.0, 159.9, 142.0, 131.7, 130.8, 129.5, 128.4, 125.9, 123.4, 122.2, 117.9, 115.9, 61.2, 61.0, 41.0, 30.9, 30.8, 30.7, 23.6, 23.5, 22.8, 20.9, 11.4

ppm. ^{195}Pt NMR (107 MHz, DMSO-d$_6$): 1634 ppm. HRMS: *m/z* calculated for $C_{26}H_{30}N_4O_{10}Pt$ $[M+Na]^+$ 776.1502, found 776.1492.

6. NMR Spectra

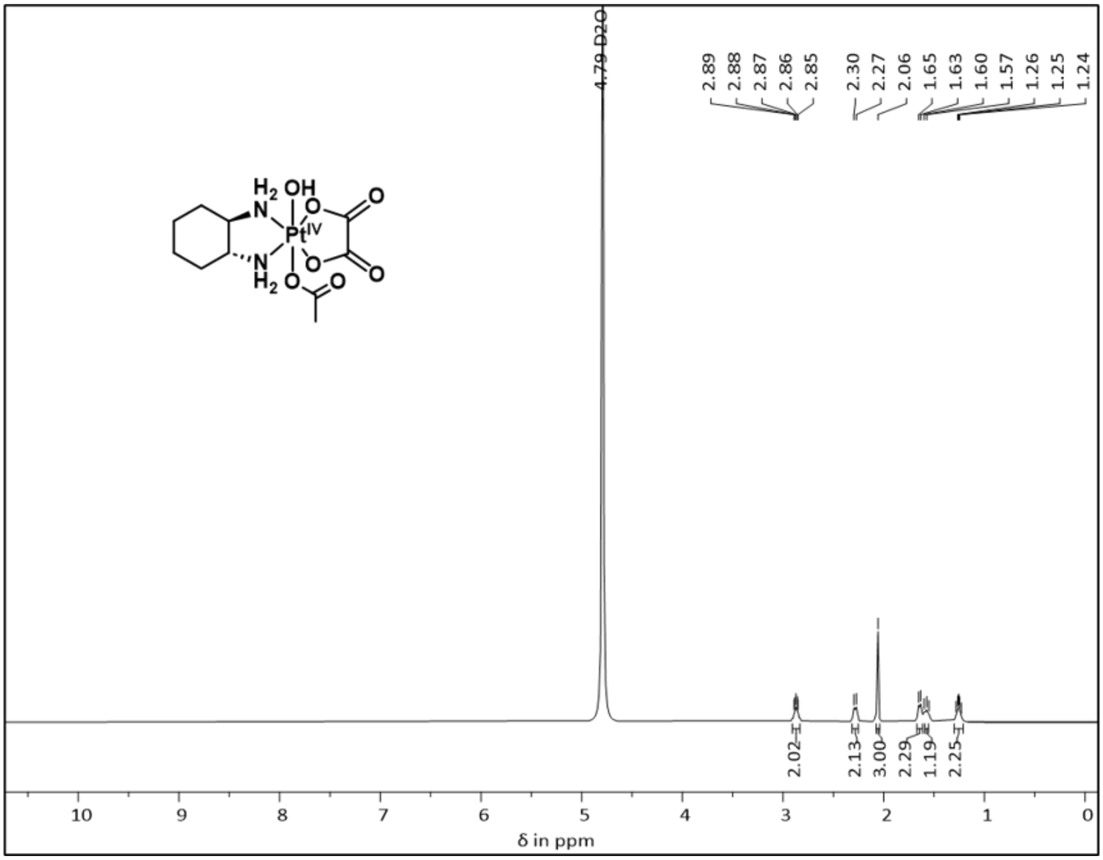

Figure S20. ^{1}H NMR (500 MHz, D_2O) of **OxPt(OH)(OAc)** (**1**)

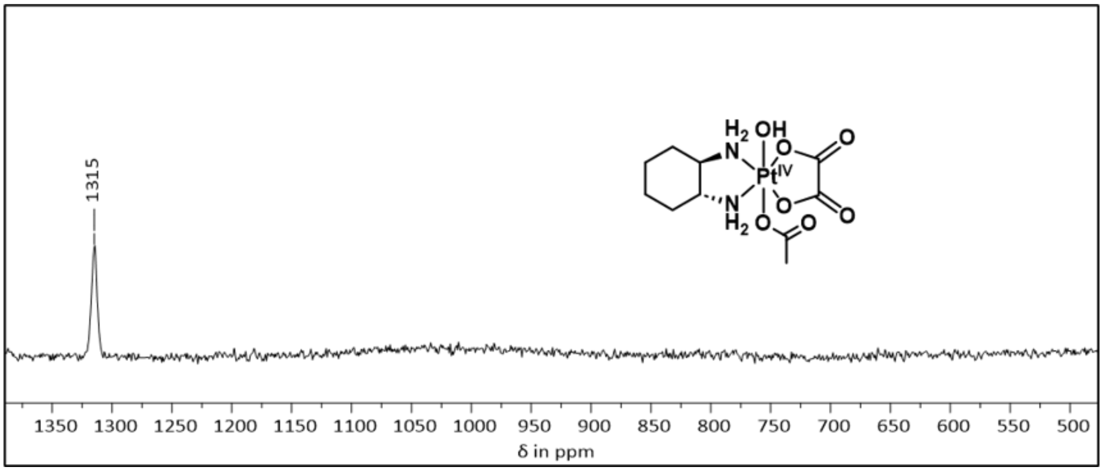

Figure S21. ^{195}Pt NMR (107 MHz, D_2O) of **OxPt(OH)(OAc)** (**1**)

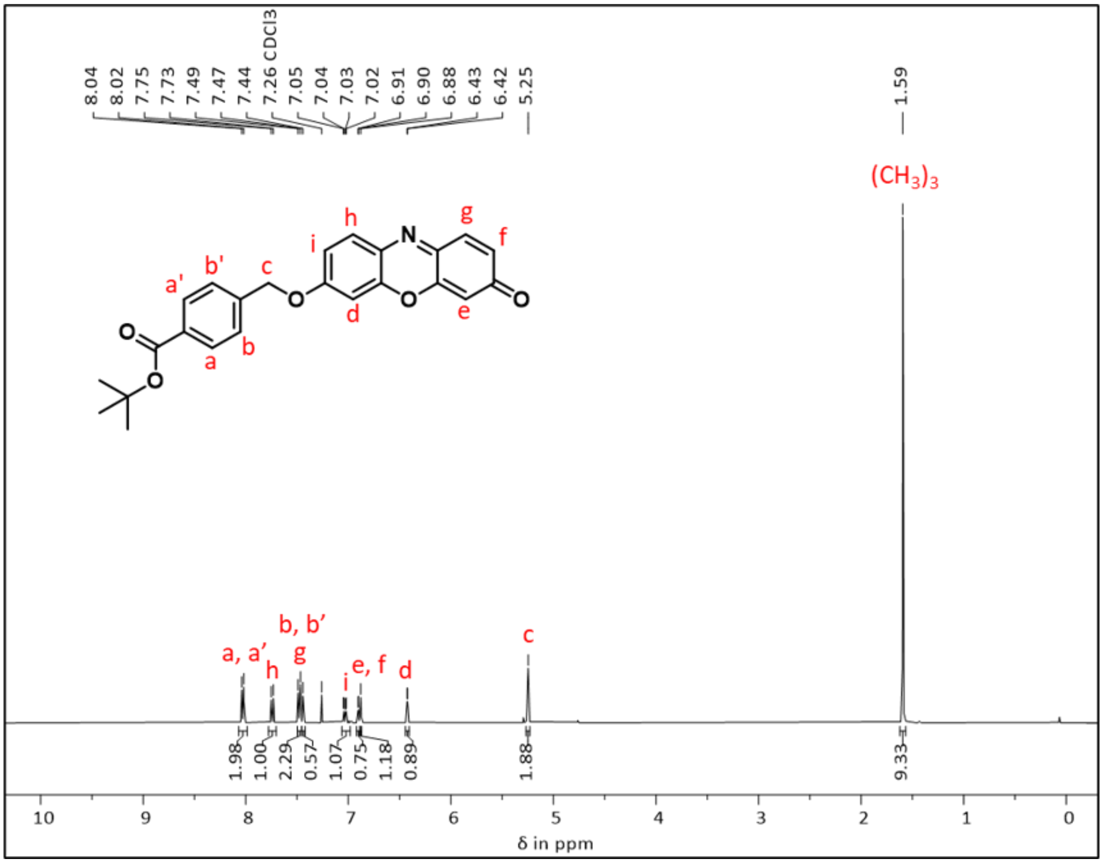

Figure S22. ^{1}H NMR (400 MHz, $CDCl_3$) of **t*ert*-butyl 4-(((3-oxo-3*H*-phenoxazin-7-yl)oxy)methyl)benzoate** (**2**)

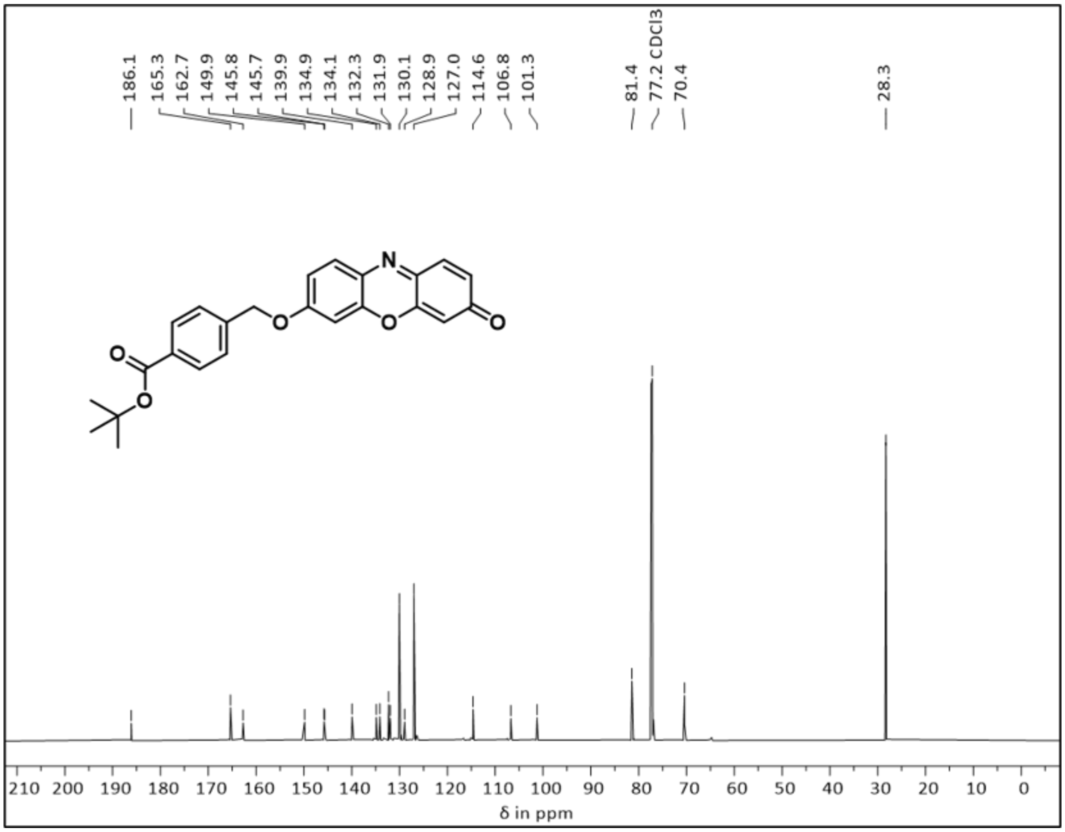

Figure S23. ^{13}C NMR (151 MHz, DMSO-d_6) of **t*ert*-butyl 4-(((3-oxo-3*H*-phenoxazin-7-yl)oxy)methyl)benzoate** (**2**)

Expanded Spectrum RT 0.20, NL 7275732.5, Peak [1], Target Mass 404.1493

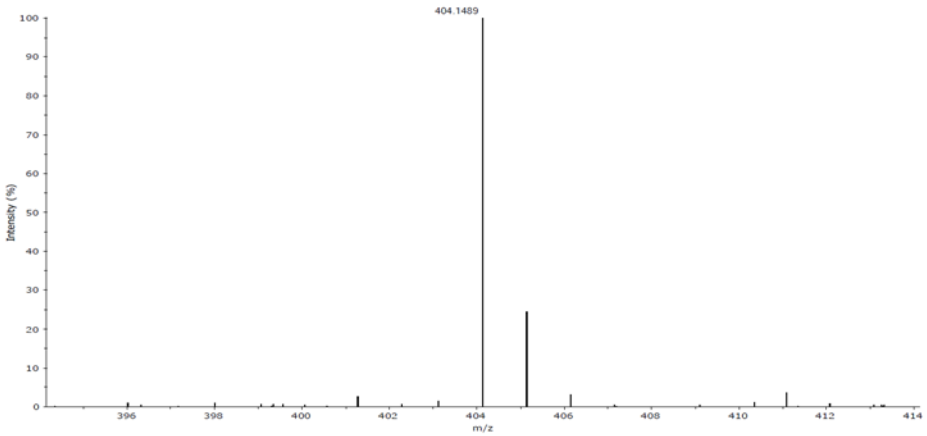

Theoretical Spectrum for C24H22NO5, Minimum Abundance 0.01%

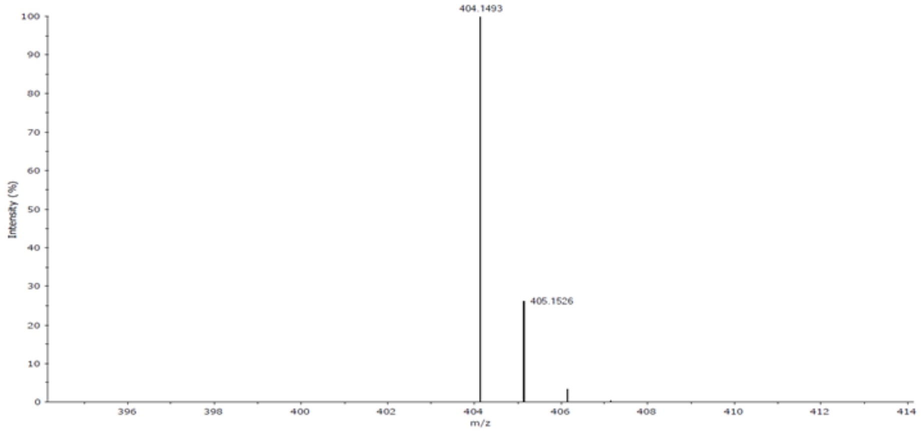

Measured Mass	Calculated Mass	Error (mDa)	Error (ppm)	Formula $[M+H]^+$	Response
404.1489	404.1493	-0.36	-0.89	$C_{24}H_{22}NO_5$	32290448

Figure S24. High-resolution mass spectrum of **t*ert*-butyl 4-(((3-oxo-3*H*-phenoxazin-7-yl)oxy)methyl)benzoate** (**2**)

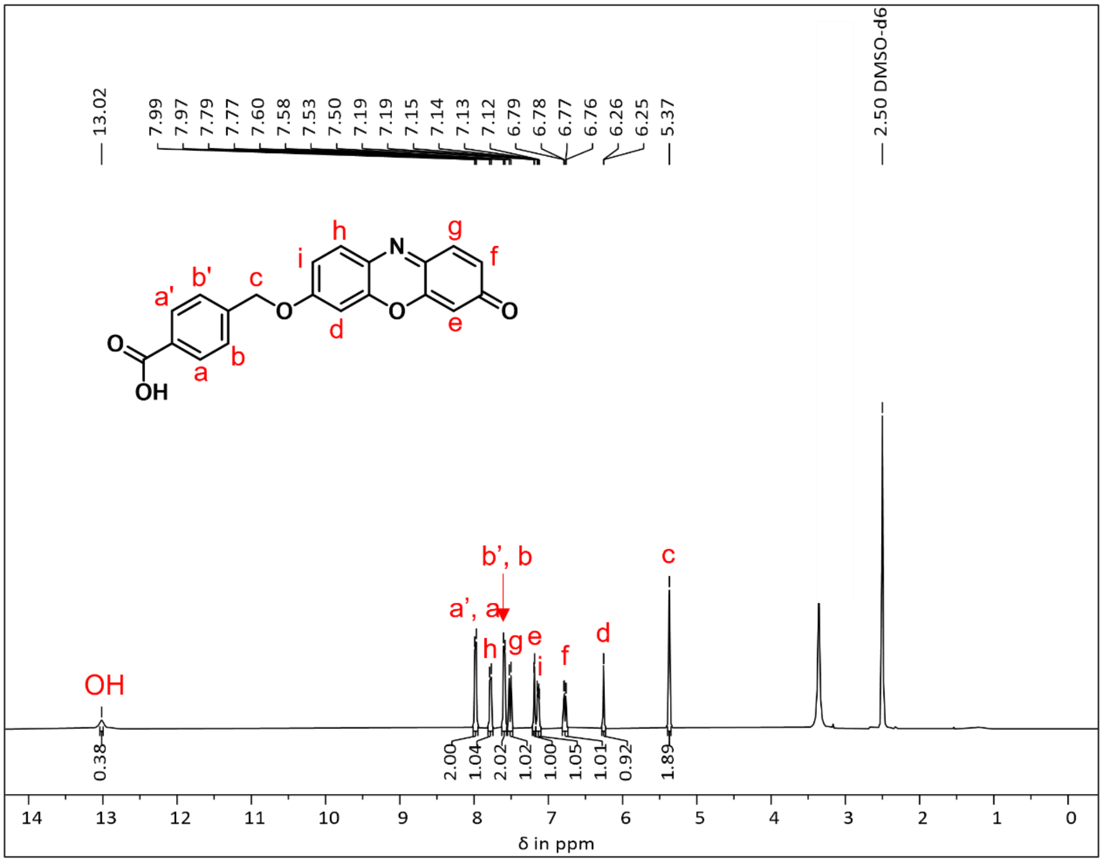

Figure S25. ^{1}H NMR (400 MHz, DMSO-d$_6$) of **4-(((3-oxo-3*H*-phenoxazin-7-yl)oxy)methyl)benzoic acid (3)**

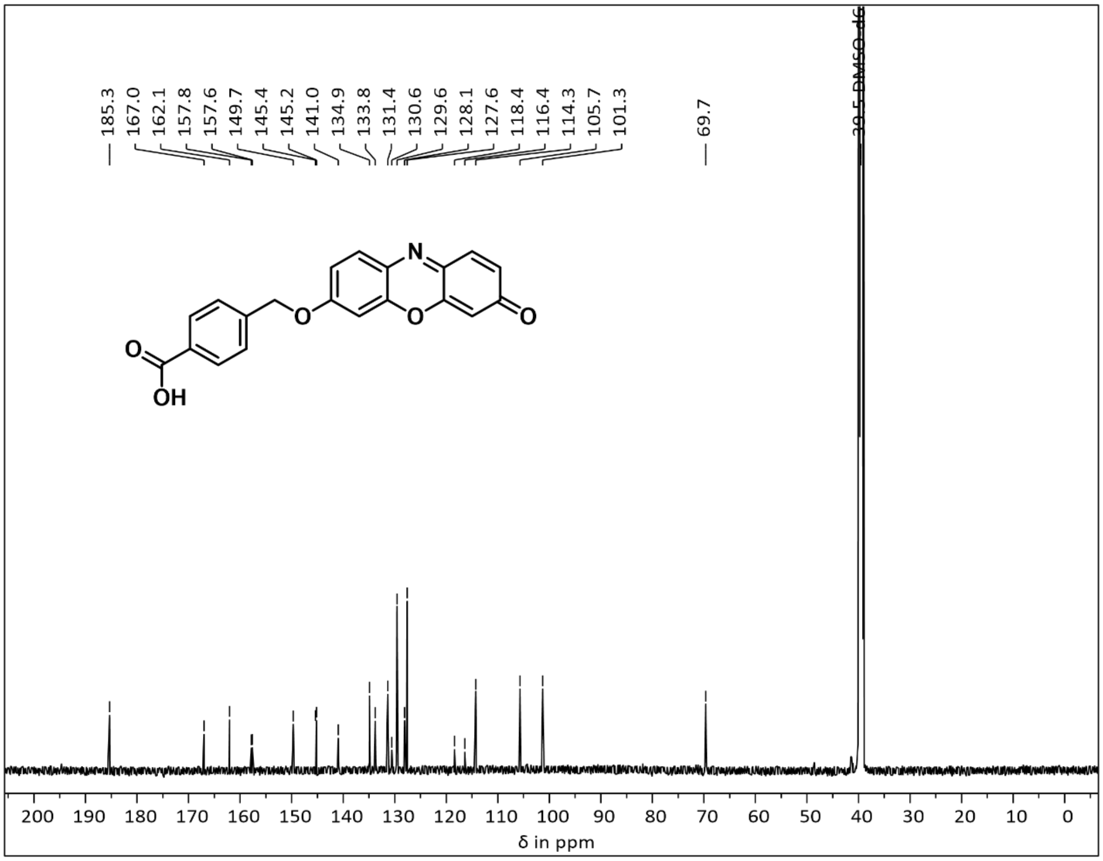

Figure S26. ^{13}C NMR (151 MHz, DMSO-d_6) of **4-(((3-oxo-3*H*-phenoxazin-7-yl)oxy)methyl)benzoic acid** (**3**)

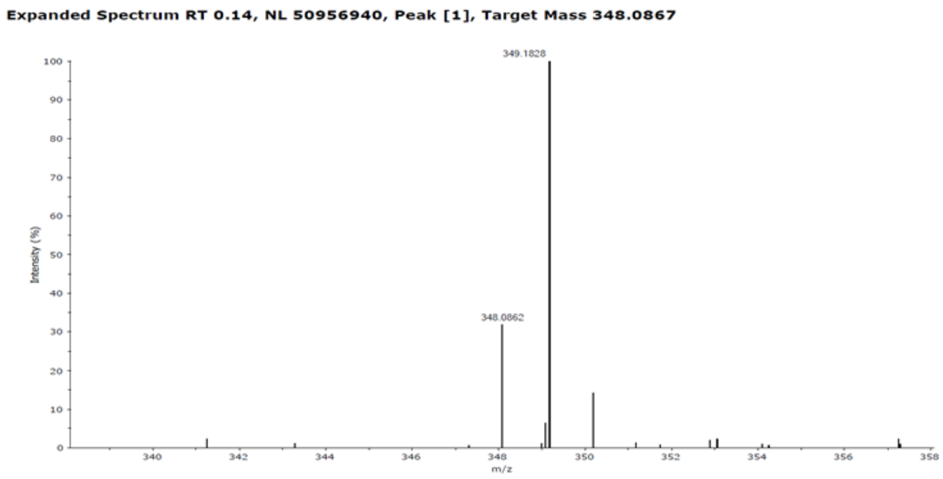

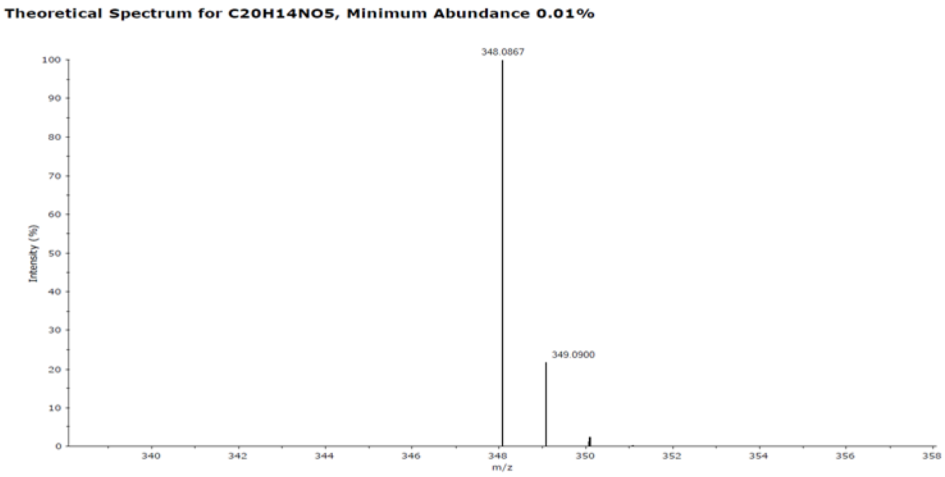

Measured Mass	Calculated Mass	Error (mDa)	Error (ppm)	Formula $[M+H]^+$	Response
348.0862	348.0867	-0.50	-1.45	$C_{20}H_{14}NO_5$	1153644

Figure S27. High-resolution mass spectrum of **4-(((3-oxo-3*H*-phenoxazin-7-yl)oxy)methyl)benzoic acid** (**3**)

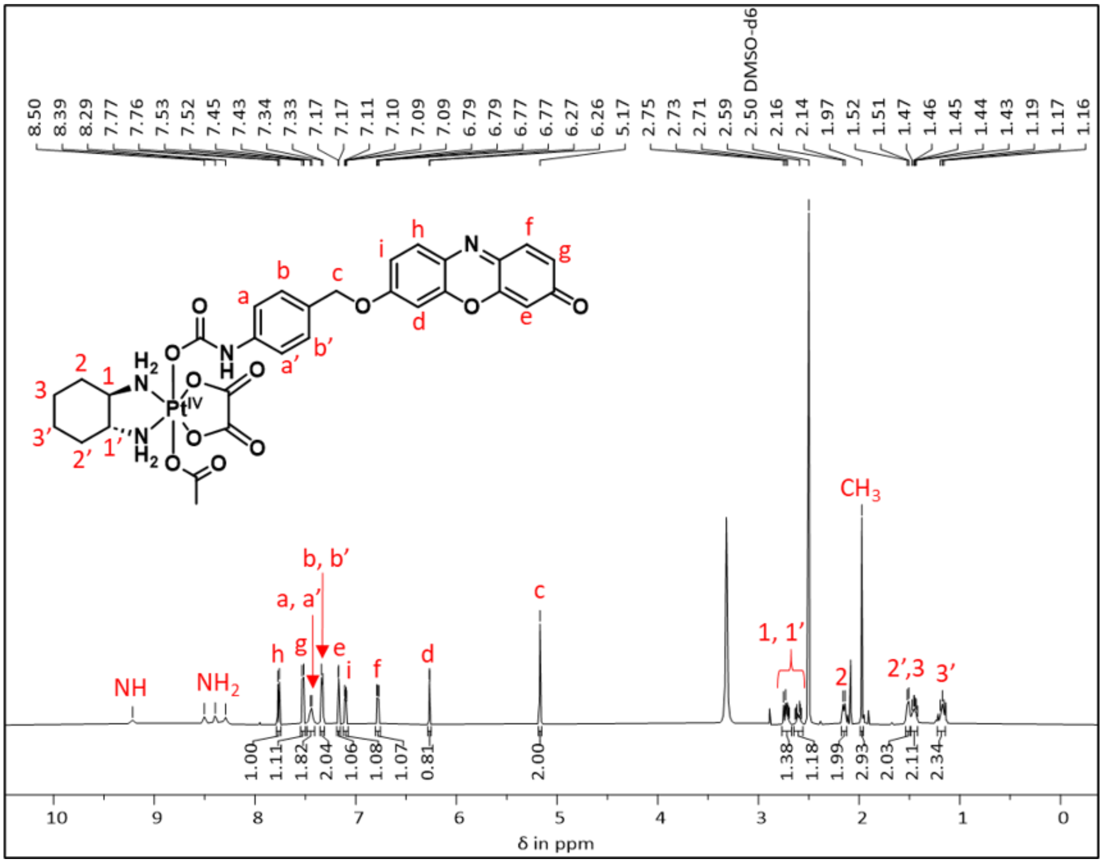

Figure S28. ^{1}H NMR (600 MHz, DMSO-d_6) of **OxaliRes** (**4**)

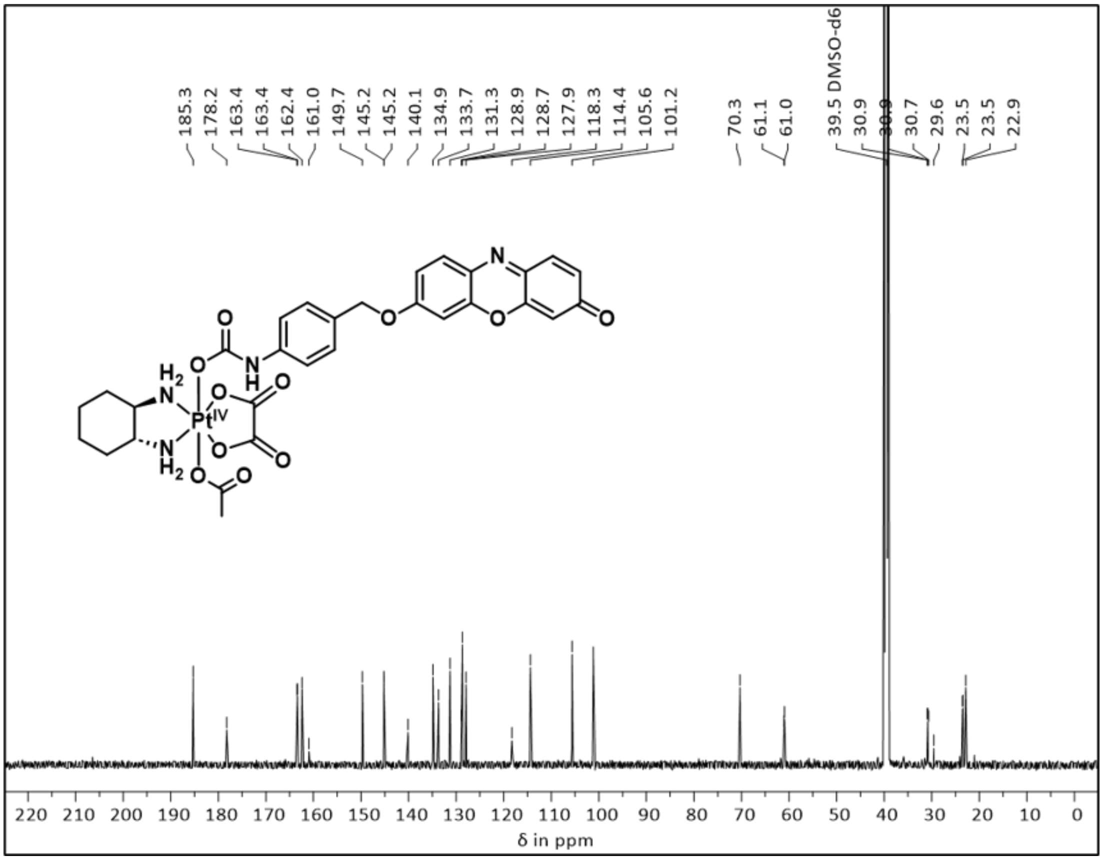

Figure S29. ^{13}C NMR (151 MHz, DMSO-d$_6$) of **OxaliRes** (**4**)

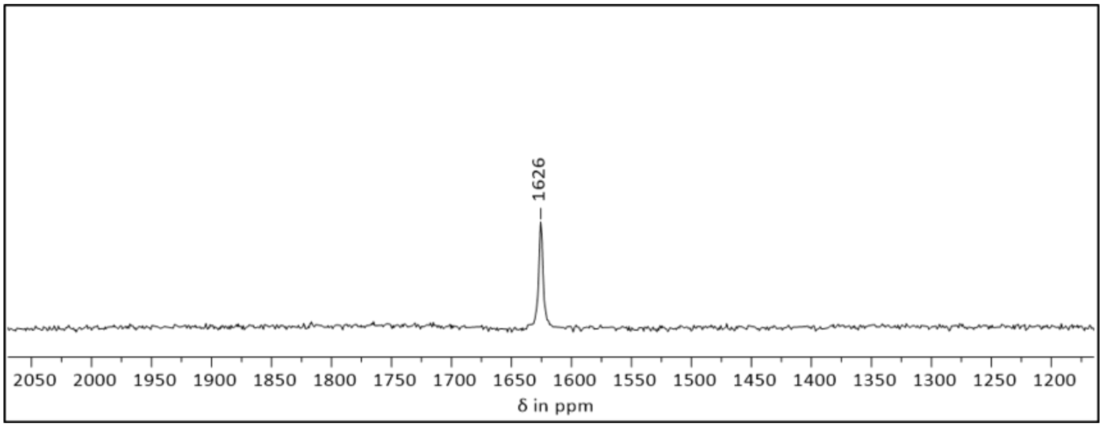

Figure S30. ^{195}Pt NMR (107 MHz, DMSO-d$_6$) of **OxaliRes** (**4**)

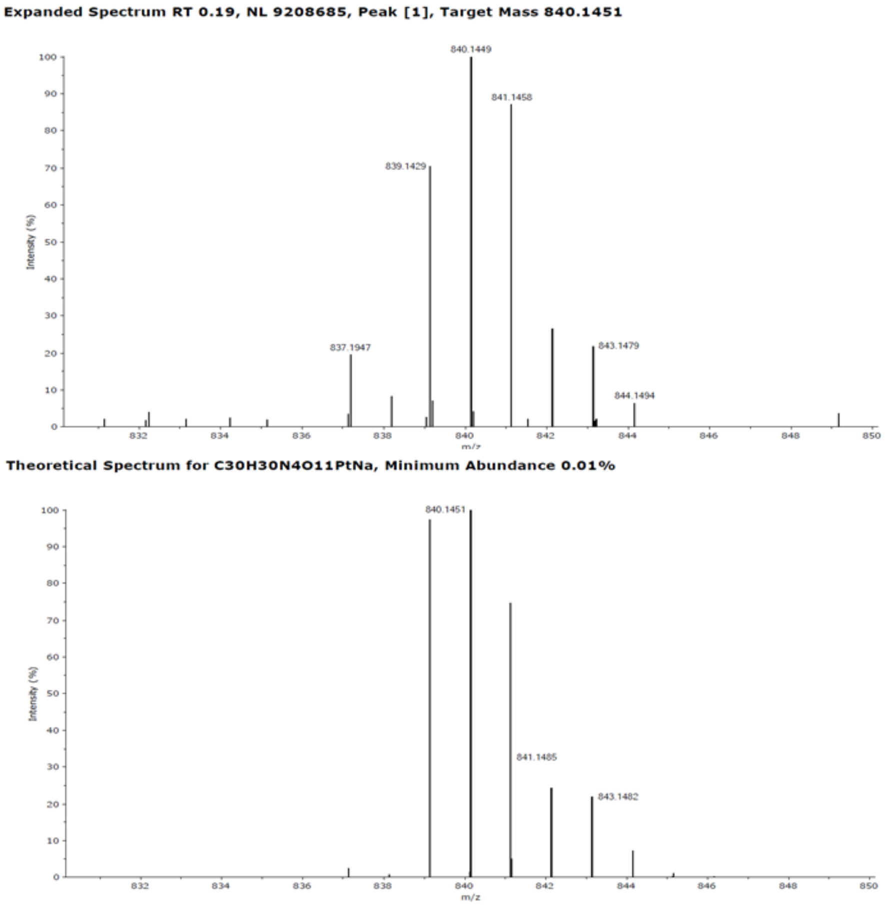

Measured Mass	Calculated Mass	Error (mDa)	Error (ppm)	Formula $[M+Na]^+$	Response
840.1449	840.1451	-0.22	-0.27	$C_{30}H_{30}N_4O_{11}PtNa$	1406938

Figure S31. High-resolution mass spectrum of **OxaliRes** (**4**)

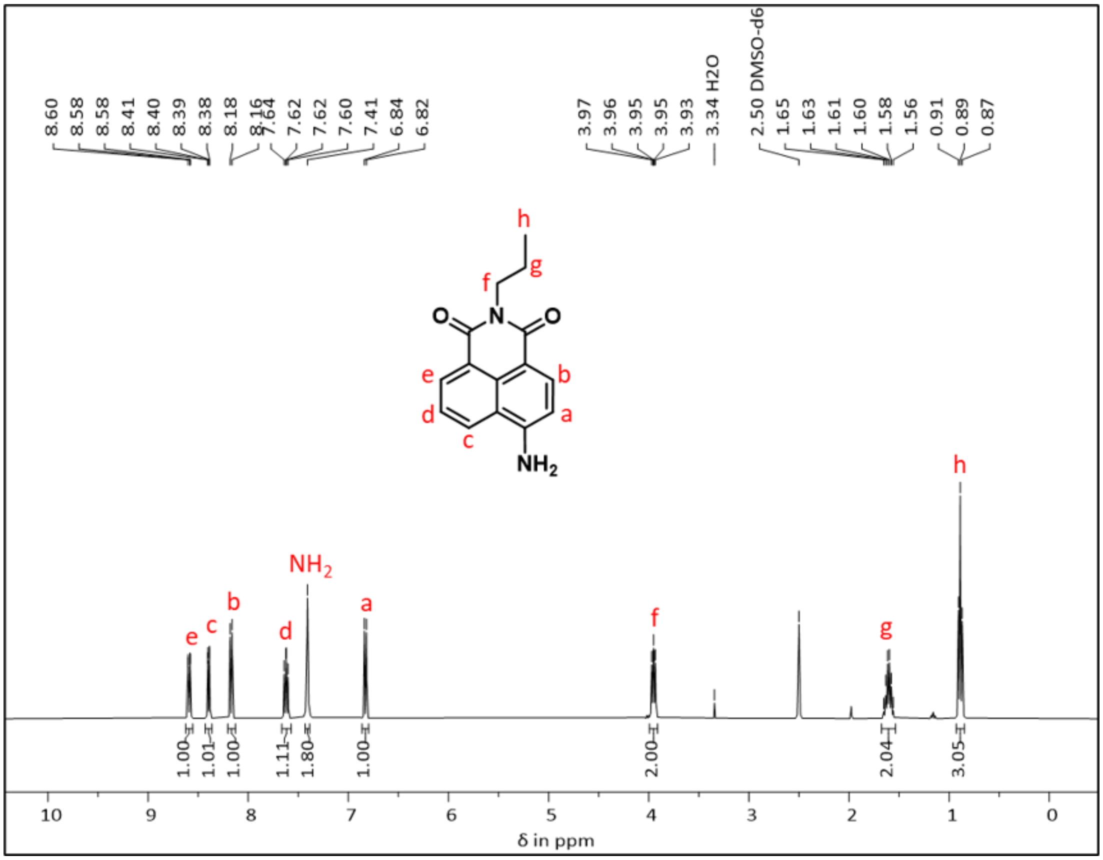

Figure S32. ^{1}H NMR (400 MHz, DMSO-d_6) of **6-amino-2-propyl-1*H*-benzo[*de*]isoquinoline-1,3(2*H*)-dione** (4-NH_2-Nap, **5**)

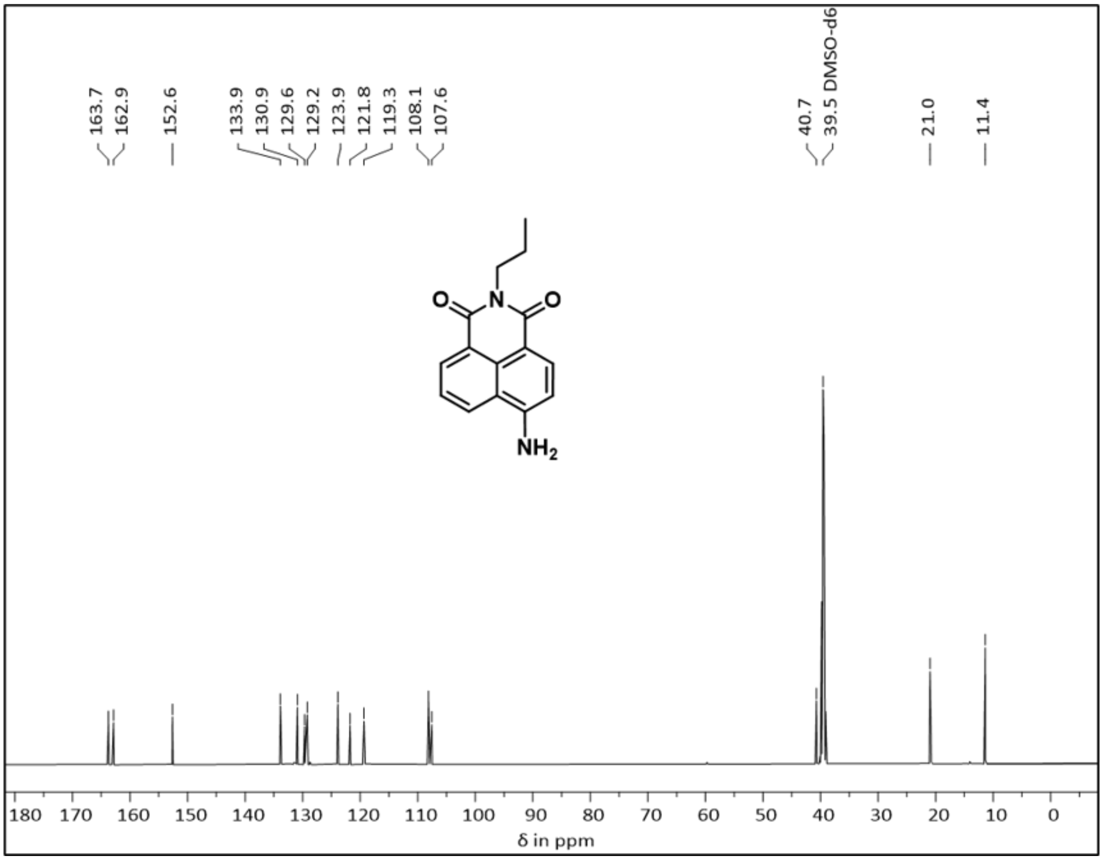

Figure S33. ^{13}C NMR (151 MHz, DMSO-d$_6$) of **6-amino-2-propyl-1*H*-benzo[*de*]isoquinoline-1,3(2*H*)-dione** (4-NH_2-Nap, **5**)

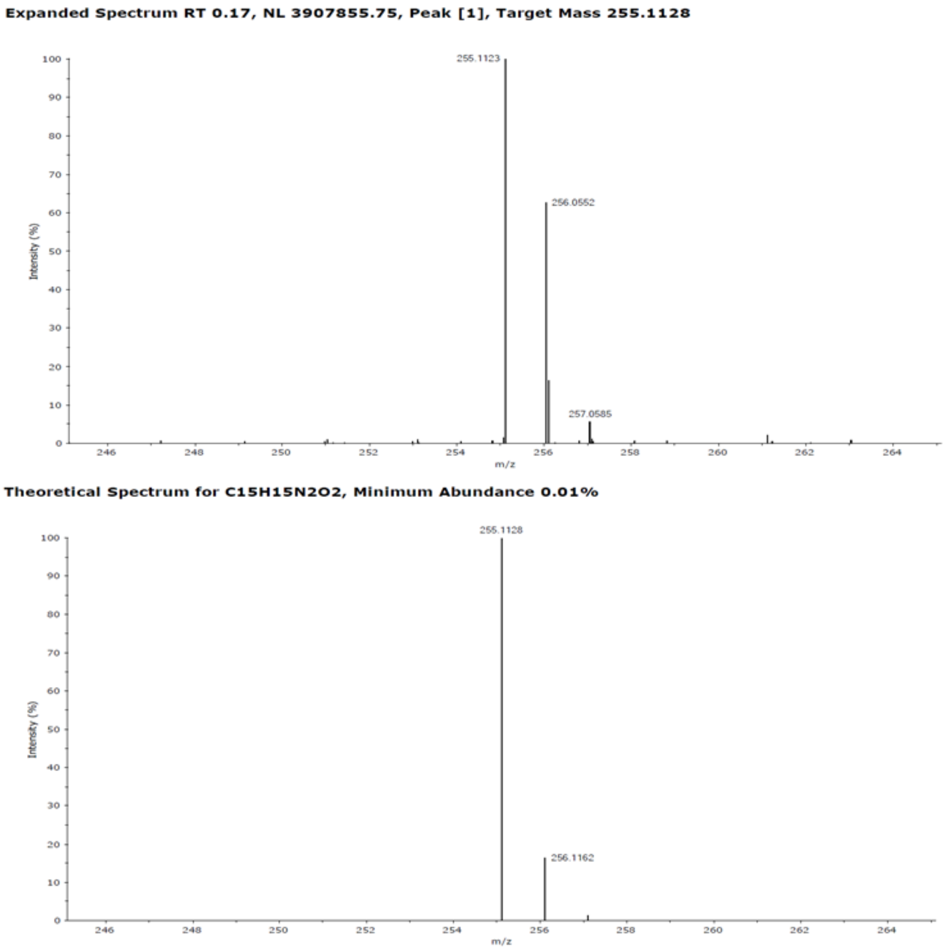

Measured Mass	Calculated Mass	Error (mDa)	Error (ppm)	Formula $[M+H]^+$	Response
255.1123	255.1128	-0.47	-1.85	$C_{15}H_{15}N_2O_2$	22878985

Figure S34. High-resolution mass spectrum of **6-amino-2-propyl-1*H*-benzo[*de*]isoquinoline-1,3(2*H*)-dione** (4-NH_2-Nap, **5**)

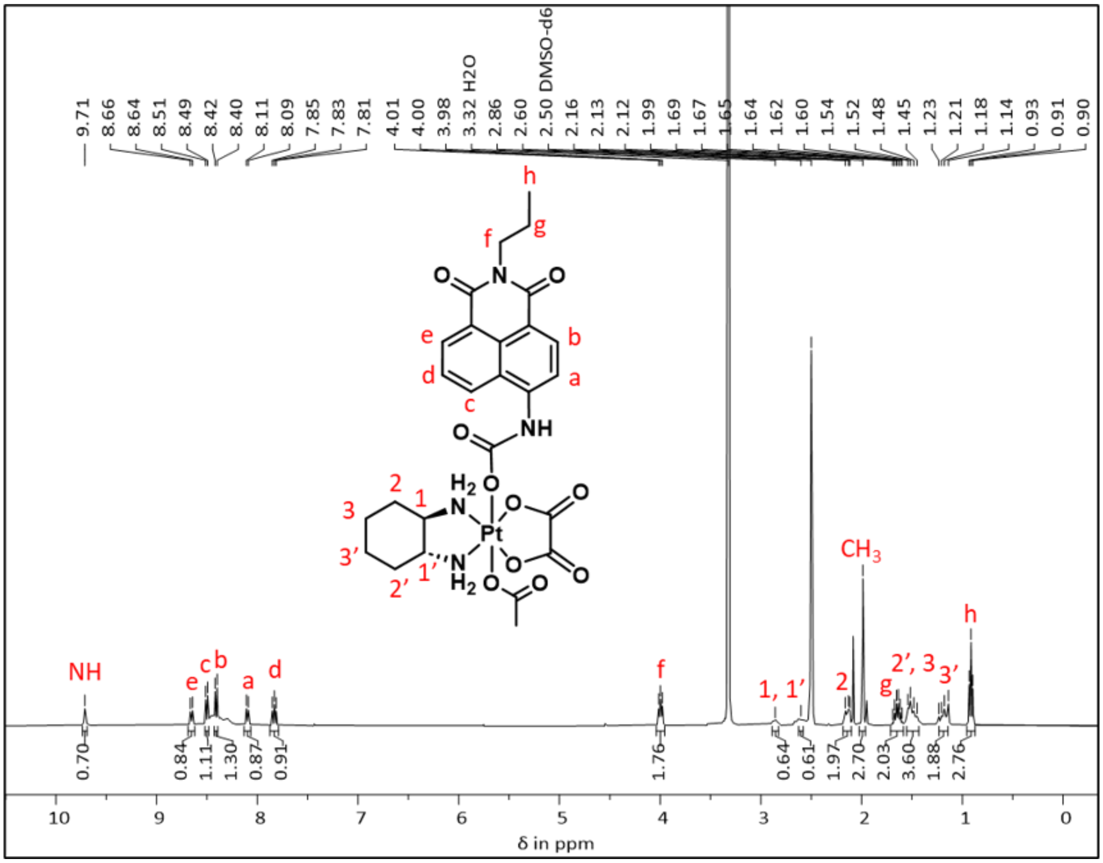

Figure S35. ^{1}H NMR (400 MHz, DMSO-d_6) of **OxaliNap** (**6**)

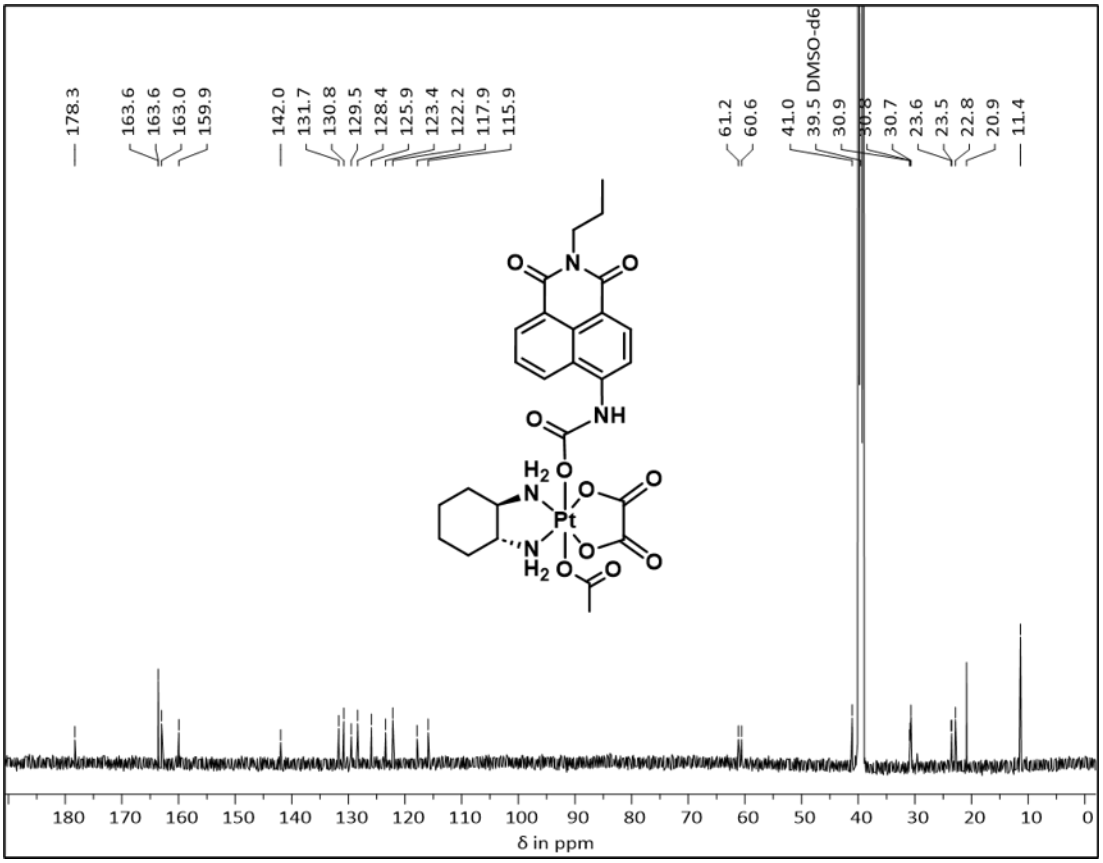

Figure S36. ^{13}C NMR (151 MHz, DMSO-d_6) of **OxaliNap** (**6**)

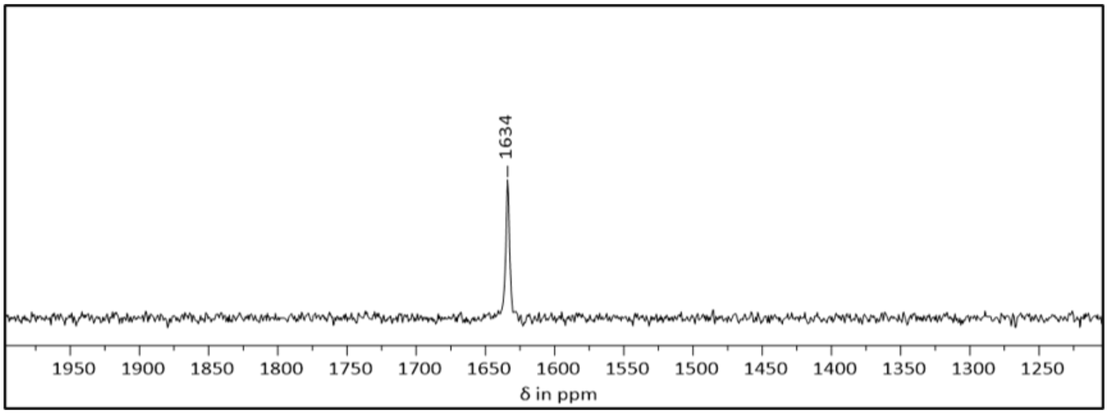

Figure S37. ^{195}Pt NMR (107 MHz, DMSO-d_6) of **OxaliNap** (**6**)

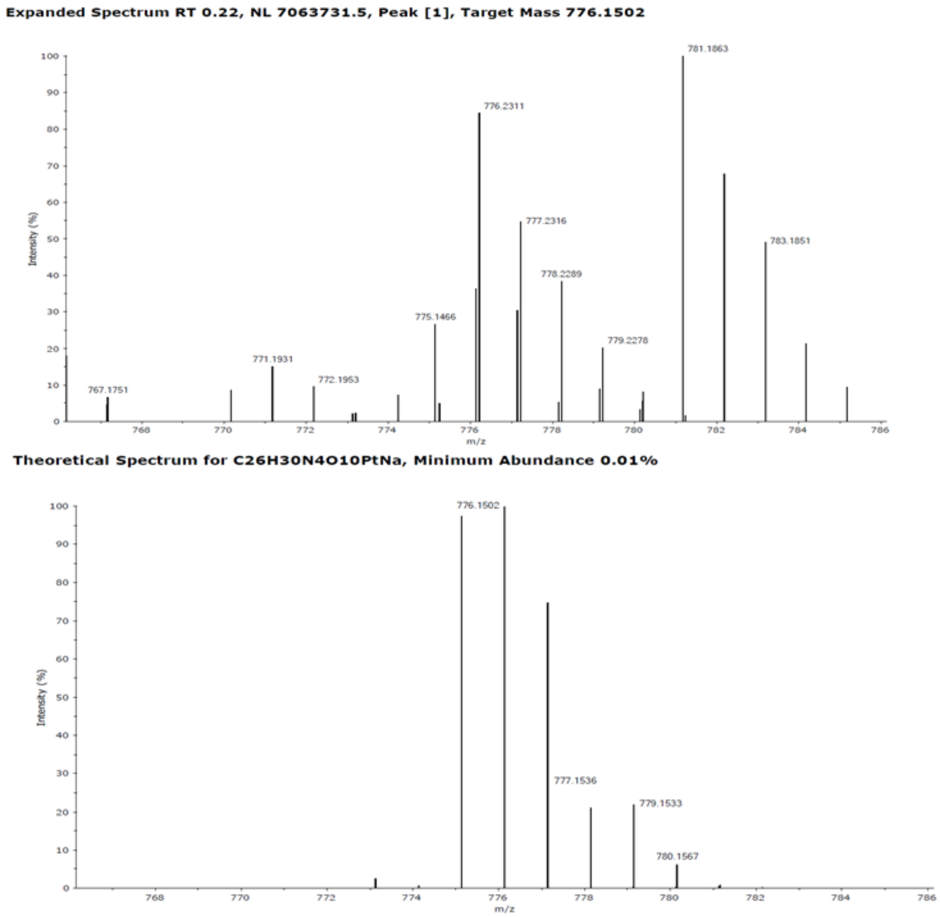

Measured Mass	Calculated Mass	Error (mDa)	Error (ppm)	Formula $[M+Na]^+$	Response
776.1492	776.1502	-1.04	-1.34	$C_{26}H_{30}N_4O_{10}PtNa$	1081681

Figure S39. High-resolution mass spectrum of **OxaliNap** (**6**)

7. References

1. G. Thiabaud, R. McCall, G. He, J. F. Arambula, Z. H. Siddik and J. L. Sessler, *Angew. Chem. Intl. Ed.*, 2016, **55**, 12626-12631.
2. W. Xuan, R. Pan, Y. Cao, K. Liu and W. Wang, *Chem. Commun.*, 2012, **48**, 10669-10671.
